# Current understanding on pathogenesis and effective treatment of glycogen storage disease type Ib with empagliflozin: new insights coming from diabetes for its potential implications in other metabolic disorders

**DOI:** 10.3389/fendo.2023.1145111

**Published:** 2023-04-21

**Authors:** Arianna Maiorana, Francesco Tagliaferri, Carlo Dionisi-Vici

**Affiliations:** ^1^ Division of Metabolism, Ospedale Pediatrico Bambino Gesù, IRCCS, Rome, Italy; ^2^ SCDU of Pediatrics, Azienda Ospedaliero-Universitaria Maggiore della Carità, University of Piemonte Orientale, Novara, Italy

**Keywords:** glycogen storage diseases, hypoglycemia, empagliflozin, neutropenia, neutrophil dysfunction, inflammatory bowel disease, autophagy

## Abstract

Glycogen storage type Ib (GSDIb) is a rare inborn error of metabolism caused by glucose-6-phosphate transporter (G6PT, *SLC37A4*) deficiency. G6PT defect results in excessive accumulation of glycogen and fat in the liver, kidney, and intestinal mucosa and into both glycogenolysis and gluconeogenesis impairment. Clinical features include hepatomegaly, hypoglycemia, lactic acidemia, hyperuricemia, hyperlipidemia, and growth retardation. Long-term complications are liver adenoma, hepatocarcinoma, nephropathy and osteoporosis. The hallmark of GSDIb is neutropenia, with impaired neutrophil function, recurrent infections and inflammatory bowel disease. Alongside classical nutritional therapy with carbohydrates supplementation and immunological therapy with granulocyte colony-stimulating factor, the emerging role of 1,5-anhydroglucitol in the pathogenesis of neutrophil dysfunction led to repurpose empagliflozin, an inhibitor of the renal glucose transporter SGLT2: the current literature of its off-label use in GSDIb patients reports beneficial effects on neutrophil dysfunction and its clinical consequences. Surprisingly, this glucose-lowering drug ameliorated the glycemic and metabolic control in GSDIb patients. Furthermore, numerous studies from big cohorts of type 2 diabetes patients showed the efficacy of empagliflozin in reducing the cardiovascular risk, the progression of kidney disease, the NAFLD and the metabolic syndrome. Beneficial effects have also been described on peripheral neuropathy in a prediabetic rat model. Increasing evidences highlight the role of empagliflozin in regulating the cellular energy sensors SIRT1/AMPK and Akt/mTOR, which leads to improvement of mitochondrial structure and function, stimulation of autophagy, decrease of oxidative stress and suppression of inflammation. Modulation of these pathways shift the oxidative metabolism from carbohydrates to lipids oxidation and results crucial in reducing insulin levels, insulin resistance, glucotoxicity and lipotoxicity. For its pleiotropic effects, empagliflozin appears to be a good candidate for drug repurposing also in other metabolic diseases presenting with hypoglycemia, organ damage, mitochondrial dysfunction and defective autophagy.

## 1 Introduction

Glycogen storage type Ib (GSDIb) is a rare inborn error of metabolism (prevalence 1:500,000) (1) caused by recessive mutations of glucose-6-phosphate translocase (*SLC37A4*), which encodes the glucose-6-phosphate transporter (G6PT) located in the membrane of the endoplasmic reticulum. The ubiquitously expressed G6PT transports glucose-6-phosphate (G6P) into the lumen of the endoplasmic reticulum where it can be hydrolyzed to glucose by glucose-6-phosphatase (G6PC1), expressed in liver, kidney and gut (2). A defect in this enzymatic complex affects glucose formation because both glycogenolysis and gluconeogenesis are inhibited, with negative effects on glycemic control. As in Glycogen storage disease type Ia (GSDIa), caused by biallelic variants in the *G6PC1* gene, mutations of *SLC37A4* result in excessive glycogen and fat accumulation in liver, kidney, and intestinal mucosa. Clinical features include hepatomegaly, hypoglycemia, lactic acidemia, hyperuricemia, hyperlipidemia, and growth retardation. Infants and children manifest hypoglycemia after 2-4 hours of fasting. Furthermore, patients with GSDIb present neutropenia, neutrophil dysfunction, and are prone to recurrent infections, ano-urogenital lesions, and inflammatory bowel disease (IBD) (3). Neutropenia can start at birth or later, it can be cyclic or permanent, with a variable clinical course (4, 5). Neutrophil count is impaired by a defective production or enhanced apoptosis. GSDIb neutrophils have a dysfunctional metabolism, which causes impaired chemotaxis, oxidative burst, and bactericidal activity (6).

## 2 Current treatment

### 2.1 Nutritional aspects

Dietary treatment is the cornerstone of management for patients with G6PC1 and G6PT deficiency, aimed to prevent hypoglycemia and minimize the risk of neurological outcome, such as developmental and motor delay, seizures and death. Nutritional therapy for GSDIa and GSDIb follows the same requirements, but further dietary manipulations are necessary in patients with GSDIb in case of IBD. Avoidance of fasting is the first line of treatment through frequent feeds of high carbohydrate low lipid diet, supplemented with cornstarch (CS). In the first year of life, nocturnal meals can be replaced by continuous enteral feeding through a nasogastric tube or gastrostomy. Surgical gastrostomy should be placed during granulocyte colony-stimulating factor (G-CSF) therapy to minimize the risk of local infection or failure in wound healing (3). American guidelines recommend to provide a nocturnal enteral glucose infusion rate of 8-10 mg glucose/kg/min in infancy, and 4-8 mg glucose/kg/min in children. Calories should be provided as 60–70% from carbohydrates, 10–15% from proteins (according to daily recommended intake), <30% from fats. CS dosage should be 1.6 g/kg (ideal body weight) every 3-4 hours in young children, and 1.7-2.5g/kg every 4-6 hours in older children and adults. Some adults may require a single dose of CS at bedtime to maintain glycemia >70 mg/dl (4 mmol/l) and lactate <2 mmol/l. However, starting from the above recommendations, nocturnal and diurnal feeding regimens are then individualized, based on glucose monitoring to achieve normoglycemia. Careful glycemic and metabolic monitoring should be performed in order to avoid overfeeding and overtreatment which lead to hyperinsulinemia, insulin resistance, obesity and nutrient deficiencies (3). In the case of IBD, continuous enteral feeding may be necessary, using an elemental diet with a polymeric formula (Modulen^®^ IBD) (7, 8), with minimum CS amount necessary to achieve glycemic and metabolic control, for the aggravative effect of CS on IBD (9).

### 2.2 Granulocyte colony-stimulating factor

The current treatment for immunological consequences is based on subcutaneous injections of G-CSF, with a starting dose of 1 μg/kg daily or alternate days, in case of severe persistent neutropenia and life threatening infection or documented IBD or severe diarrhea requiring hospitalization or disrupting normal life (10). The G-CSF dose should be gradually increased to obtain a neutrophil count of 0.5-1.0×10^9^/l (3). However, G-CSF can improve neutrophil count but is ineffective on neutrophil dysfunction (6). Furthermore, it may worse splenomegaly and osteopenia (11) and increase the risk of malignancies (acute myeloid leukemia and myelodysplasia) (11–13). Evidences about the efficacy of G-CSF in treating GSDIb patients resulted from retrospectives studies and case reports (3).

## 3 Pathophysiology

**Figure 1:**
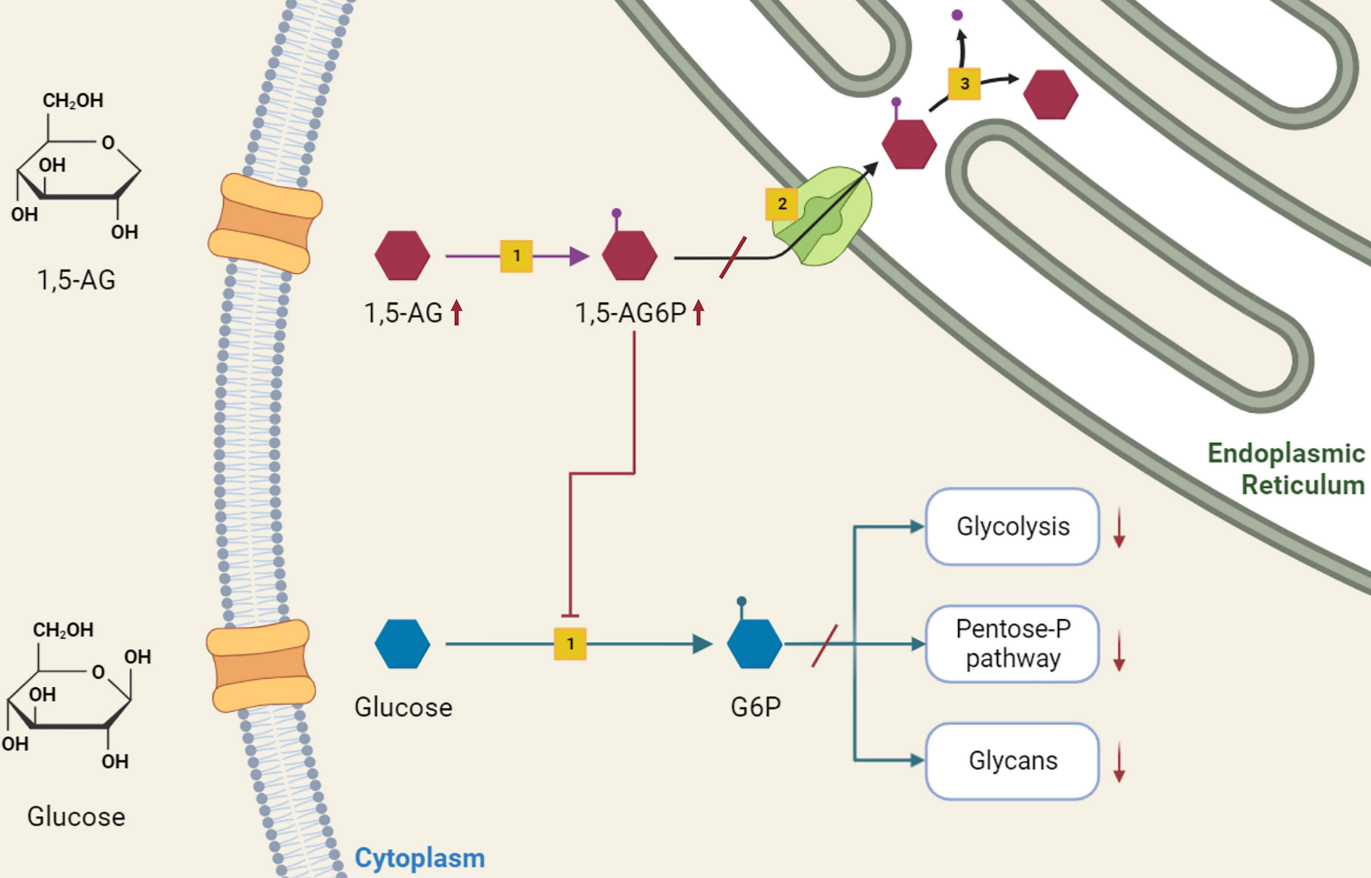
Role of 1,5-anhydroglucitol-6-phosphate accumulation in neutropenia and neutrophil dysfunction of G6PT deficiency. 1,5-AG, normally present in blood, enters into neutrophils where is slowly phosphorylated by a side activity of hexokinases [1] into 1,5-AG6P. G6PT [2] transports 1,5-AG6P into the endoplasmic reticulum, where it is dephosphorylated by G6PC3 [3]. In GSDIb patients, the deficiency of G6PT leads to 1,5-AG6P accumulation, which in turn inhibits hexokinase activity. This inhibition affects the conversion of glucose into G6P, interfering with downstream metabolic pathways of glycolysis, pentose-phosphate pathway and glycosylation, with consequent impaired neutrophil survival, respiratory burst and protein glycosylation.

The accumulation of 1,5-anhydroglucitol-6-phosphate (1,5-AG6P) within granulocytes was recently recognized as the cause of neutrophil dysfunction in GSDIb (14). The formation of 1,5-AG6P results from the action of hexokinases on 1,5-anhydroglucitol (1,5-AG, 1-deoxyglucose). This polyol, physiologically present in the blood, is a structural dietary analogue of glucose, which originates from dietary sources and has a constant blood concentration maintained by a renal clearance (15). However, it can also derive from an endogenous intestinal production (14). From neutrophil cytosol, G6PT transports 1,5-AG6P to endoplasmic reticulum, where is reconverted into 1,5-AG by glucose-6-phosphatase catalytic subunit 3 (G6PC3), homologous to G6PC1. In individuals lacking either G6PT (causing GSDIb) or G6PC3 (causing neutropenia type IV) 1,5-AG6P accumulates in neutrophils cytosol, where it inhibits the conversion of glucose into G6P by hexokinases. The reduced G6P lowers the glucose amount for glycolysis and pentose-phosphate pathway. In turn, the decreased ATP production from glycolysis affects neutrophil survival, and the defective NADPH production from the pentose-phosphate pathway impairs the respiratory burst. Also, the reduced UDP-glucose availability interferes with glycan formation and protein glycosylation (14) (**Figure 1**).

### 3.1 In vitro studies

Veiga da Cunha et al. used CRISPR-Cas9 to generate human HAP1 cell lines deficient in G6PC3 or G6PT, to investigate whether their deficiency leads to the accumulation of 1,5-AG6P. Addition of 1,5-AG to the culture media led to a dose-dependent accumulation of 1,5-AG6P in G6PC3- or G6PT-deficient cell lines, while its levels in wild-type cells remained ∼100 times less. This demonstrated that 1,5-AG is phosphorylated into 1,5-AG6P and confirmed the hypothesis that both G6PC3 and G6PT are essential for the hydrolysis of 1,5-AG6P (14). Furthermore, consistent with the idea that 1,5-AG6P might inhibit the first step of glycolysis by inhibiting hexokinase 1 (as previously showed in brain (16)), the glycolytic intermediates (glucose-6-P, fructose-6-P, ribose-5-P, 6-phosphogluconate and fructose-1,6-bisphosphate) resulted significantly reduced.

Toxicity of 1,5-AG was first demonstrated in immortalized neutrophil progenitor cell line from G6PC3-deficient mice, in whom physiological concentrations of 1,5-AG in the culture medium caused massive accumulation of 1,5-AG6P, reduced G6P concentration, reduced glycolysis, reduced survival and cellular death (14).

### 3.2 In vivo studies

Leukocytes from 3 neutropenic patients with deficiency of G6PC3 or G6PT showed a concentration of 1,5-AG6P 500 times higher than controls. Furthermore, the concentration of 1,5-AG6P was higher in neutrophils than peripheral blood mononuclear cells in the same patient. These results were consistent with the idea that G6PT and G6PC3 are involved in 1,5-AG6P metabolism in human neutrophils and, when they are defective, 1,5-AG6P accumulates in the cytosol and inhibits hexokinases. Therefore, the consequent neutropenia is a metabolite-repair deficiency, caused by a failing to remove the nonclassical metabolite 1,5-AG6P (14).

## 4 Empagliflozin

Empagliflozin, an inhibitor of the renal sodium-glucose cotransporter type 2 (SGLT2), is an antidiabetic drug that inhibits the renal glucose reabsorption, causing the excretion of glucose and other sugars in diabetic and normoglycemic patients. 1,5-AG is normally filtered in the glomeruli and reabsorbed in the proximal renal tubules by specific active transporters (17). Since the reabsorption of 1,5-AG is competitively inhibited by glucose (18), conditions of increased glucosuria (such as uncontrolled diabetes or the use of SGLT2 inhibitors) lead to increase the urinary 1,5-AG excretion and lower its blood concentrations (6, 19–22).

Veiga da Cuhna et al. demonstrated that treatment with empagliflozin in G6PC3 deficient mice aimed to lower their blood level of 1,5-AG, decreased intracellular levels of 1,5-AG6P and normalized the neutrophil count. Conversely, treatment with 1,5-AG increased levels of 1,5-AG6P and provoked a neutrophil maturation arrest with accumulation of apparent promyelocytes (14).

### 4.1 Evidences from glycogen storage disease type Ib

After these results, the authors administered empagliflozin as off-label use in 4 GSDIb patients (3 children and 1 adult) with incomplete response to G-CSF to treat neutropenia and neutrophil dysfunction (6). Repurposing of empagliflozin therapy at the dosage of 0.3-0.7 mg/kg/day determined a decreasing of serum 1,5-AG and neutrophil 1,5-AG6P levels within one month, and patients showed clinical improvements with decreased symptoms of recurrent infections, mucosal lesions, and resolution of IBD. No symptomatic hypoglycemia was observed during treatment. G-CSF was discontinued in two patients and could be tapered by 81% and 57% in the remaining two patients, respectively. Neutrophil count increased and stabilized in all patients. Normalization of neutrophil oxidative burst, protein glycosylation, chemotaxis and bactericidal activity indicated the correction of neutrophil dysfunction (6).

G-CSF discontinuation after empagliflozin therapy was later reported in a 35-year old GSDIb patient with a concomitant improvement of wound healing and symptoms of IBD, without side effects (23).

Rossi et al. treated with empagliflozin a 14-year old GSDIb patient with severe IBD: he presented an improvement of clinical symptoms and stool frequency within the first week of therapy and normalization of the Pediatric Crohn disease activity index (PCDAI) within the first month. A significant decrease in disease activity was confirmed at the abdomen magnetic resonance imaging after 3 months of treatment, data confirmed on histology at 5.5 months. G-CSF dosage was reduced. Ameliorated metabolic control was noted in the present case. Stable lactate concentrations, normalization of triglycerides (TG) and recovery from hyperuricemia were reported. Liver and kidney function were normal and no adverse event was described. Healing from IBD might have yielded a positive effect on metabolic control, increasing intestinal glucose absorption and affecting the gut microbiota (24, 25), and certainly improved the overall psychosocial well-being of patient (24).

Another report confirmed that empagliflozin ameliorated neutropenia, neutrophil dysfunction, and IBD symptoms in a child, reducing serum 1,5-AG, with unchanged occasional hypoglycemia (26). Similar results were described in a 35-year old patient, who could reduce G-CSF dosage. No hypoglycemic event was reported (18).

Kaczor and coll. reported a series of 4 children treated with empagliflozin, confirming its positive effect on decreasing infections, improving IBD and wound healing. G-CSF was withdrawn or reduced. One patient experienced a single hypoglycemic episode during treatment, related to a delay of the nocturnal meal intake (27).

Grunert and coll. reported the favorable use of empagliflozin in a pregnant GSDIb patient: since the glycemic values were very unstable in the first weeks of gestation, the medication was reduced, causing a recrudescence of oral mucosal lesions, thereby the previous dose was restored. After the caesarean section, breastfeeding followed regularly. However, even the other reported patient, who was not treated with empagliflozin until childbirth, experienced recurrent mild hypoglycemia requiring glucose supply during pregnancy. G-CSF was discontinued in both patients, with normalization of neutrophil count and wound healing (28).

A group of 8 treated GSDIb pediatric patients with a median age of 7.8 years (range 1.5-15.8) was recently reported, with a cumulative treatment time more than 12 years: beneficial effects were showed on neutrophil function, anemia and growth. Plasma 1,5-AG levels reduced by a median of 78%. Half of the patients presented hypoglycemic episodes following the start of treatment, and one patient had hypoglycemic seizures: by lowering empagliflozin dosage and increasing carbohydrate intake, hypoglycemia resolved. Patients manifested an improvement in median Z-score for height and BMI, and a stable metabolic control (29).

Bidiuk et al. described 2 GSDIb adult siblings: in one suffering from heart failure, the treatment was associated to an improvement of cardiomyopathy, allowing the reduction of cardiological therapy and the withdrawn of eplerenone. Symptomatic hypoglycemia was not reported. The treatment with empagliflozin allowed to reduce the G-CSF dosage and the number of medications for concomitant clinical conditions. In his brother, who also suffered from HLA-B27 positive arthritis and sinus tachycardia, empagliflozin allowed corticosteroid withdrawal and antiarrhythmic therapy was tapered, without alterations in glycemic control (30).

Hexner-Erlichman and coll. reported 2 patients (17-year and 8-year old) who received empagliflozin for 18 months and 24 months, respectively. The treatment was safe and effective, resulting in normalization of neutrophil count and function, and yielded the resolution of mucosal lesions and improvement of IBD with discontinuation of G-CSF. No hypoglycemic event was recorded in both patients (31).

Using untargeted metabolomics profiling, Tallis et al. monitored empagliflozin treatment in an 11-year old girl, following the reduction of 1,5-AG levels; concurrently, neutrophil counts and function improved, as well as her IBD symptoms with reduction of G-CSF dose. Empagliflozin dose was adjusted because of arthralgia, but no other side effects were reported. Fasting tolerance improved (32). Untargeted metabolomics also showed modifications of other metabolic pathways of lipids metabolism, as previously reported in GSDIb subjects (33), and empagliflozin-related improvement in other parameters of metabolic control, such as urate and urate intermediates (32).

Empagliflozin effects on neutrophil dysfunction, glycemic and metabolic control from the above-described case reports were detailed in **Table 1**.

**Table 1:** Empagliflozin effects in GSDIb patients reports.

Study	Gender Age	Empagliflozin final dose	Treatment duration	1,5-AG^§^	Neutrophil count/function	Skin-mucosal lesions	IBD	Hypoglycemic events	Lactate	Uric acid	TG	Micro-albuminuria	Other treatments	Notes
Wortmann et al., 2020 (6)	♀ 21y	0.4 mg/kg/d (10 mg x 2/d)	288 d	↓	↑/↑	↓	↓	1 mild (2.7 mml/L) 9h after 1^st^ dose, no other	na	na	na	na		Increased appetite and oral feeds
	♀ 2y	0.3 mg/kg/d (5 mg/d)	217 d	↓	↑/↑	↓	–	none	na	na	na	na		Increased appetite and oral feeds
	♂ 6y	0.7 mg/kg/d (10 mg x 2/d)	246 d	↓	↔/↑ ↓G-CSF	↓	↓	none	na	na	na	na	Sulfasalazine; antiepileptics; G-CSF (↓)	From drip feeding to oral feeds portions; improvement of glycemic control, no more hypoglycemia induced seizures
	♀ 2y	0.5 mg/kg/d (7.5 mg/d)	191 d	↓	↑/↑	↓	↓	Occasional (2,8 – 3.9 mmol/L) asymptomatic, reduced frequency	na	na	na	na	Sulfasalazine (stop)	↑ interval between meals; improvement of glycemic control
Grunert et al., 2020 (23)	♀ 34y	0.4 mg/kg/d (10 mg x 2/d)	>80 d	na	↔/↑ Stop G-CSF	↓	↓	none	na	na	na	–	G-CSF (stop); pancreatin, budesonide; vitamin E; loperamide (stop); 5-aminosalicylic acid	Stool frequency decreased; CDAI 398➔184
Mikami et al., 2021 (26)	♀ 30m	0.5 mg/kg/d	>60 d	↓	↑/↑	–	↓	Hypoglycemia (<2.8 mmol/L) before breakfast, frequency not changed by therapy	na	↓	↓	na	na	
Rossi et al., 2021 (24)	♂ 14y	0.4 mg/kg/d (10 mg x 2/d)	232 d	↓	↑/na ↓ G-CSF	–	↓	1^st^ week: < 3.3 mmol/L on 20/32 low-glucose events at FGM, asymptomatic; 2–4 asymptomatic mild events/month (2.7–3.3 mmol/L)	↔, in range	↑, in range*	↑, in range	–	G-CSF (↓); allopurinol (stop)	Juvenile idiopathic arthritis. From exclusive tube feeding, restart oral feeds portions (d164)
Kaczor et al., 2022 (27)	♂ 17.5y	0.3 mg/kg/d	1.5 y	na	↑/na Stop G-CSF	↓	↓	none	↑, in range	↓, in range	↔, in range	–	G-CSF (stop); zoledronic; mesalazine	Stool frequency decreased; reduced hospitalization
	♂ 13y	0.4 mg/kg/d	1 y	na	↑/na Stop G-CSF	↓	↓	none	↓	↓, in range*	↓*	–	G-CSF (↓); mesalazine; allopurinol; fenofibrate; febuxostat	Stool frequency decreased; reduced hospitalization
	♀ 9y	0.4 mg/kg/d	1 y	na	↔/na ↓ G-CSF	↓	↓	One episode (1.44 mmol/L) related to delayed night meal	↔, in range	↓, in range	↔, in range	–	G-CSF (stop); mesalazine	Stool frequency decreased; reduced hospitalization
	♀ 17m	0.4 mg/kg/d	6 m	na	↑/na Stop G-CSF	↓		none	↑	↓, in range	↔, high	–	G-CSF (stop)	
Grunert et al., 2022 (28)	♀ 34y	na		na	↑/na	↓	–	na	na	na	na	–	G-CSF (stop); vitamins; L-thyroxine	Pregnancy, Empagliflozin started after delivery
	♀ 35y	2 x 10 mg/d		na	↑/na	↓	↔	Severe hypoglycemia (≈ 2.2 mmol/L) three times throughout pregnancy	na	na	na	–	G-CSF (stop); Phenprocoumon➔ enoxaparin	Empagliflozin started during pregnancy; breastfeeding
Tallis et al., 2022 (32)	♀ 11y	0.6 mg/kg/d (10 mg x 2/d)	22w		↔/↑ ↓ G-CSF	na	↓	After attempts to space fasting intervals	↑, in range	↓	na	–	G-CSF (↓)	Generalized arthralgia after dose escalation; PCDAI 35➔27.5; increase fasting time
Halligan et al., 2022 (29)	7y	0.2 mg/kg/d	1.7y	↓	↓/na Stop G-CSF	na	↔	4/8 patients experienced hypoglycemia upon start of treatment; 1/8 significant symptomatic hypoglycemic events including an overnight hypoglycemic seizure	na	↓	↓	na	2/8 allopurinol (1 stop, 1 ↓)	3♂,5♀; 3 of 4 pts studied had baseline neutrophil dysfunction, normalized after therapy Anemia resolved in all patients; PUCAI score improve in 4/6 patients. One patient had loss of previously established metabolic control, (elevated urate, triglycerides, and pre-feed lactate levels)
	15.2y	0.1 mg/kg htwt/d	1.3y	↓	↓/na	na	↓		na	↓	↔	na		
	8.6y	1.3 mg/kg htwt/d	1.83y	↓	↑/na Stop G-CSF	na	↓		na	↔	↑	na		
	4.3y	0.4 mg/kg htwt/d	2.3y	↓	↑/na Stop G-CSF	na	↓		na	↓	↑	na		
	15.4y	0.2 mg/kg htwt/d	0.8y	↓	na/na	na	na		na	na	na	na		
	15.8y	0.1 mg/kg htwt/d	1.8y	↓	↑/na	na	↓		na	↓	↔	na		
	1.5y	0.2 mg/kg htwt/d	0.6y	↔	↔/na	na	na		na	↓	↓	na		
	6.8y	0.2 mg/kg htwt/d	2.1y	↓	↔/na	na	↑		na	↓	↑	na		
Bidiuk et al., 2022 (30)	♂ 29y	20 mg/d	12m	na	↑/na ↓ G-CSF	↓	↓	none	↔, high	↔, high	↑ *	+	G-CSF (↓); metoprolol (↓); ramipril (↓); eplerenone (stop); fenofibrate (start) [other therapies, not specified]	Heart failure; reduction of the number of medications
	♂ 28y	20 mg/d	12m	na	↑/na ↓ G-CSF	–	↔	none	↔, high	↔, high	na	+	G-CSF (↓); metoprolol (↓); ivabradine (↓); methylprednisolone (stop); allopurinol; sulfasalazine (↓); fenofibrate [other therapies, not specified]	HLA-B27 positive arthritis; wheel-chair-bound; sinus tachycardia; reduction of the number of medications
Makrilakis et al., 2022 (18)	♀ 32y	0.4 mg/kg/d (25 mg/d)	>300 d	↓	↑/na	na	↓	none	na	↔, in range	↔, in range	na	G-CSF (↓); allopurinol; mesalazine (stop); denosumab	CDAI 356➔52
Hexner-Erlichman et al, 2022 (31)	♀ 17.5y	0.65 mg/kg/d	18m		↑/na	↓	↓	none	na	na	na	+	G-CSF (stop); ACEi; prednisone (stop); sulfasalazine; fibrate; omega-3 fatty acids	Glomerular and tubular dysfunction; chronic pancreatitis
	♂ 8.5y	0.5 mg/kg/d	24m		↑/na	↓		none	na	na	na	–	Endocrine and exocrine pancreatic replacement therapy	

^§^Reference values different for every work, see references.; *Concomitant therapy.

ACEi, ACE inhibitor; CDAI, Crohn’s Disease Activity Index; mg/kg htwt, milligrams per kilogram of height weight; na, not available; PCDAI, Pediatric Crohn’s Disease Activity Index; PUCAI, Pediatric Ulcerative Colitis Activity Index; TG, triglycerides; ↑, increase; ↓, decrease; ↔, stable; +, pre-existing, no data on empagliflozin; -, absent.

Furthermore, the results of a multicenter study on the safety and efficacy of empagliflozin in GSDIb patients was recently reported. Clinical data from 112 subjects were collected through an international retrospective questionnaire from 24 countries. Empagliflozin was started at a mean age of 12.8 years (range 0-38), majority <18 years. Mean duration of treatment was 10.1 months (range <1-27), and the median dose was 0.35 mg/kg/day (range 0.1-0.9). Patients showed an improvement of mucosal lesions, recurrent infections and symptoms of IBD. The 5% discontinued G-CSF and 17% tapered the dosage. Hypoglycemia was reported in 18% of patients and was not related to the dose frequency. Lactic acidosis was reported in 5% of patients (5 children, 1 adult), requiring hospitalization. One of the adult individuals also had two hospitalizations in intensive care unit for ketoacidosis, during gastroenteritis and dehydration (34).

### 4.2 Evidences from neutropenia type 4

The first evidence for the use of empagliflozin in G6PC3 deficiency were recently described in 3 patients: a 30-year old woman with neutropenia, recurrent infections and inflammatory enterocolitis (35) and 2 children with recurrent infections (17). Improvement of neutropenia and colitis manifestations as well as of the quality of life was reported. In these patients, the dosage of empagliflozin (0.03-0.28 mg/kg/day) able to decrease plasma 1,5-AG was lower than GSDIb patients: a possible explanation could be that patients with G6PC3 deficiency do not take CS, an important source of 1,5-AG (36), therefore they may need a lower dose of empagliflozin to correct neutrophil dysfunction (35). Furthermore, mutation in other sodium-glucose transporters, such as SGLT5, might explain higher urinary excretion of 1,5-AG, milder neutropenia and a better responsiveness to empagliflozin in some patients (17).

### 4.3 Evidences from diabetes

#### 4.3.1 Metabolic effects

A prospective trial (NCT01248364) was conducted to evaluate the metabolic effects of empagliflozin after a single dose and after four weeks of treatment in patients with type 2 diabetes (T2DM). Postprandial glucose and insulin AUC significantly decreased, whereas the glucagon increased. Fasting endogenous glucose production increased, and tissue glucose disposal was reduced. Chronic dosing shifted the substrate utilization from carbohydrates to lipids (37). Subsequent studies confirmed that the glucosuric effects of empagliflozin resulted in a decrease of plasma glucose levels (38–41), glycemic spikes and HbA1c levels (42). However, empagliflozin has pleiotropic effects, such as reducing insulin levels, insulin resistance, glucotoxicity and lipotoxicity, and increasing glucagon secretion and ketone bodies production (42, 43). Furthermore, empagliflozin improves redox state and oxidative stress, inhibiting reactive oxygen species (ROS) production, reducing the activity of pro-oxidant agents, and improving mitochondrial function (44). Of note, all these processes are implicated in the pathogenesis of metabolic, cardiovascular (CV), renal and neurological consequences of diabetes (42, 45). Particularly, in diabetes and obesity the excessive circulating free fatty acids accumulate in the adipose tissue mainly as TG. When fat storage exceeds subcutaneous and visceral adipose tissue capacity, ectopic fat accumulates in other tissues, such as skeletal muscle and myocardium, liver and pancreatic β-cells, with progressive impairment of mitochondrial function. This process of lipotoxicity generates a chronic low-grade inflammation and an insulin resistance state, which lead to impaired glucose tolerance, dyslipidemia, and hypertension (46, 47). Furthermore, the non-fasting hyperlipidemia causes endothelial stress through the production of inflammatory cytokines and oxidative stress agents, and the reduction of endothelial nitric oxide synthase activity (48, 49). Empagliflozin exerts protective effects from lipotoxicity, decreasing lipid accumulation in visceral fat (43) and promoting weight loss (50), reducing inflammation and oxidative stress (42).

Uric acid is another well-established pro-inflammatory mediator (51). Empagliflozin has been demonstrated to lower uric acid in T2DM patients with a likely positive effect on low-grade inflammation (50, 52). Several mechanism have been hypothesized, including the inhibition of renal tubular uric acid transporters in response to glucosuria and the inhibition of xanthine oxidase for uric acid production (53).

Conversely, the action of insulin on low-grade inflammation is more discussed, with different reports suggesting a time and dose-dependent activity (54, 55). However, in a recent study, patients on therapy with SGLT2-inhibitors (SGLT2-I) showed decreased levels of insulin and uric acid, and lower levels of IL-6, a marker of low-grade inflammation associated to diabetes complications (52).

#### 4.3.2 Cardiac effects

Hyperglycemia along with inflammation and oxidative stress are the main cause of vascular dysfunction and CV disease in diabetes. The efficacy of empagliflozin in reducing CV risk and macrovascular complications in the diabetic population have been demonstrated in several pioneering clinical trials, such as EMPA-REG OUTCOME, EMPEROR-Reduced and EMPEROR-Preserved (56–58). Its diuretic and hypotensive effects are hemodynamic mechanisms (43) which involve several pathways. Glucosuria leads to a negative energy balance, which generates a ‘fasting-mimicry’ condition. Although the amount of SGLT2-I-induced urinary loss of glucose may result arduous to estimate, evidences from a caloric deficit come from patients with familial renal glucosuria type 1 (*SLC5A2* deficiency), in whom glucosuria can range up to 150 g/1.73 mq/day (59), which corresponds to ~600 kcal/day. The negative energy balance yields activation of the SIRT1/AMPK signaling pathway, with consequent suppression of Akt/mTOR signaling (60) and positive effect on metabolism, mitochondrial function and oxidative stress.

Oxidative stress from dysfunctional mitochondria plays a pivotal role in diabetic cardiomyopathy. Remarkably, in diabetes the increased free fatty acids accumulate in cardiomyocytes as lipid droplets containing TG, diacylglycerol (DAG) and ceramides which contribute to the development of left ventricular hypertrophy and cardiac dysfunction (lipotoxicity) (61), and to the exacerbation of insulin resistance (43). The latter reduces the glucose utilization and overbalances the lipidic metabolism towards fatty acids accumulation, which leads to progressive mitochondrial dysfunction and consequent increased oxidative stress. This process in turn aggravates the diabetic cardiomyopathy and myocardial dysfunction (62).

**Figure 2:**
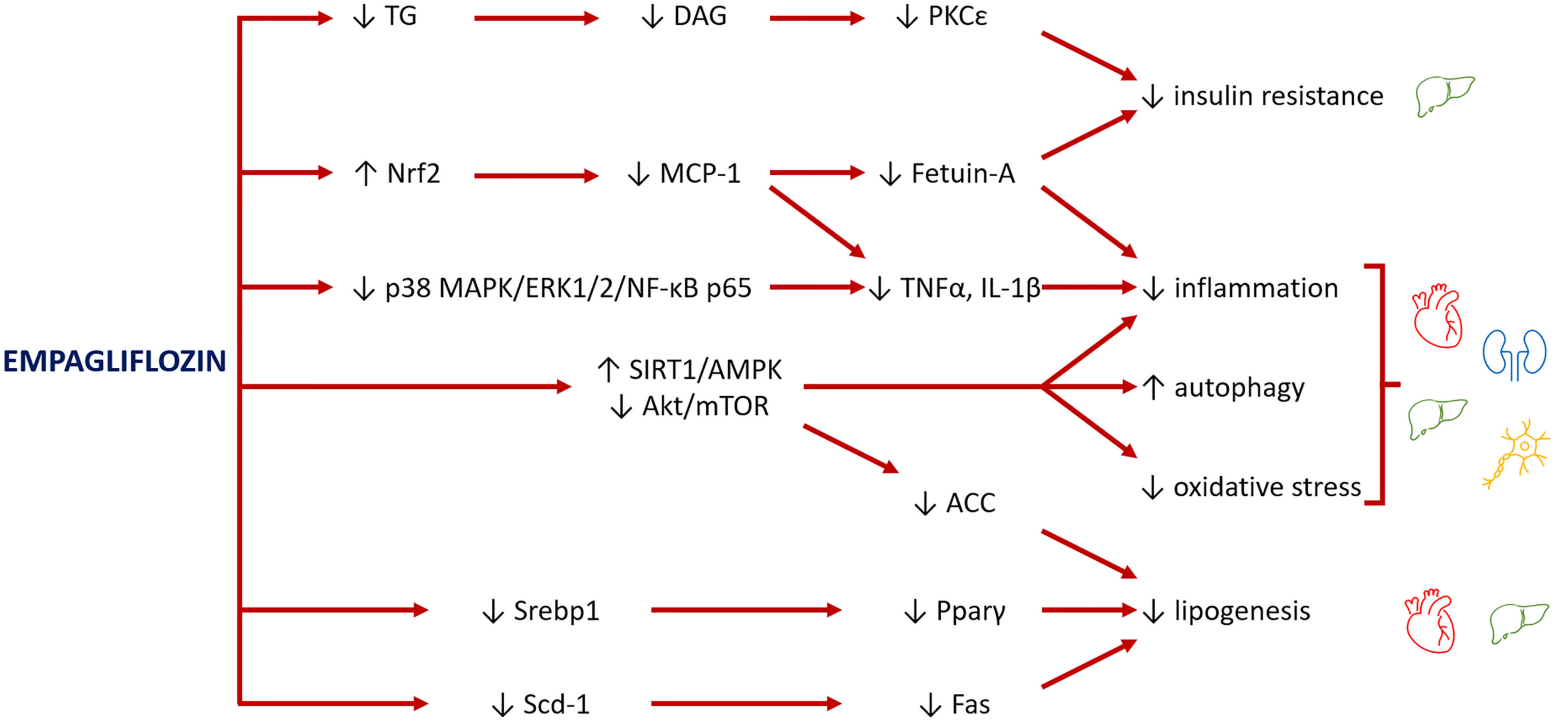
Major pathways involved in the rescue of organ damage from empagliflozin. Empagliflozin reduces insulin resistance by decreasing the levels of triglycerides (TG) and their lipotoxic intermediates diacylglycerols (DAG), lowering the inhibitory effect of PKCϵ on insulin receptor. Empagliflozin also increases levels of Nrf2, a mediator between lipid metabolism and antioxidant defense, with consequent inhibition of MCP-1 and fetuin-A and reduction of inflammation and oxidative stress. A further mechanism to inhibit inflammation is through suppression of p38 MAPK/ERK1/2/NF-κB p65 expression. The main role of empagliflozin is activation of SIRT1/AMPK and inhibition of Akt/mTOR pathways, which reduce inflammation, improve mitochondrial function and autophagy, and decrease oxidative stress. Finally, empagliflozin reduces the levels of transcription factors (Srebp1 and Pparγ) and the expression of enzymes (Scd-1, Fas), leading to a reduction of lipogenesis, with benefits especially for the heart and liver.

Empagliflozin has been proved to activate AMPK in mice cardiomyocytes and restore energy levels (63), via acetyl coenzyme A carboxylase (ACC) inhibition, which enhances fatty acid oxidation and increases ATP generation (64). Furthermore, AMPK activation plays a main role in stimulating autophagy and in turn having cardio- and mitochondrial protective effects (60, 65) (**Figure 2**). Of note, mitochondrial autophagy (mitophagy) which is regulated by several signaling pathways, including AMPK-mTOR (43), plays a cardioprotective role through the clearance of abnormal mitochondria, thus preventing the oxidative stress and reducing the myocardiocytes apoptosis (66).

Furthermore, empagliflozin down-regulates the Na^+^/H^+^ antiport, reducing the cytoplasmic sodium and calcium levels and increasing the mitochondrial calcium concentrations, which in turn enhances the ATP synthesis and improves the cardiac contractile activity (67), regardless the antihyperglycemic effect (68).

In addition, studies conducted on mice and rats models suggested protective effects of SGLT2-I against the development of fibrosis in different types of cardiopathy and nephropathy (69–71).

#### 4.3.3 Renal effects

The SGLT2-I drugs reduce the reabsorption of glucose and sodium, leading to a diuretic osmotic effect. Differently from loop diuretics, SGLT2-I causes a greater reduction of fluids in the interstitial compartment, resulting in a lower depletion of circulating volume and minor effect on tissue perfusion (72). In type 1 diabetes patients with hyperfiltration (GFR > 135 ml/min), empagliflozin has been documented to reduce the intraglomerular pressure and the glomerular filtration rate (GFR) of – 30 ml/min, through the sodium reabsorption (73). Also T2DM patients are at risk of developing diabetic nephropathy. Hyperfiltration is the first stage of nephropathy, which may progress towards glomerular fibrosis, renal failure and end-stage renal disease (74). Several trials showed that empagliflozin yielded significant renoprotective effects in subjects with T2DM (75–79) and end-stage kidney disease (80). Furthermore, it lowered the estimated GFR and reduced the urine albumin-to-creatinine ratio in both microalbuminuric and macroalbuminuric ranges (81, 82). Therefore, the 2021 ADA guidelines recommended SGLT2-I in T2DM patients with nephropathy to lower the risk of renal failure, major CV events and heart failure hospitalization (83).

Inhibition of SIRT1/AMPK signaling and autophagy is also involved in the pathogenesis of the glomerular and tubular lesions in diabetic nephropathy, particularly in the tubular accumulation of advanced glycation end products and in the podocyte injury (84–86). SGLT2 expression is stimulated by glucose, acting as a sensor of energy abundance. Its activity is inversely related to SIRT1 expression and autophagic activation in the kidney (87). Therefore, pharmacological modulation of SGLT2 may have a main role in modifying the energy/redox sensing molecules to stimulate autophagy (60) (**Figure 2**). Remarkably, there are evidences that empagliflozin ameliorated diabetic tubulopathy and renal fibrosis by controlling autophagy in mice (88).

#### 4.3.4 Hepatic effects

Non-alcoholic fatty liver disease (NAFLD) is a wide spectrum of chronic liver disorders, ranging from steatosis to non-alcoholic steatohepatitis, fibrosis, and cirrhosis (89). The association between NAFLD and T2DM increases the risk of advanced liver damage (90). Furthermore, T2DM patients with NAFLD are at higher CV risk, suggesting that these conditions share common pathogenic mechanisms, such as low-grade systemic inflammation and oxidative stress (91).

In a prediabetic rat model, empagliflozin had ameliorative effects on hepatic lipid metabolism regardless obesity, by decreasing lipogenesis, fetuin-A and inflammation (92, 93) (**Figure 2**). Fetuin-A is an hepatokine which represents an important link between adipose tissue, liver and skeletal muscle (92). It promotes insulin resistance by a direct action on the insulin receptor (93) and stimulates the low-grade inflammation binding the toll-like receptor 4, thus producing a lipid-induced pro-inflammatory response (94). By decreasing serum concentration of fetuin-A, empagliflozin likely exerted an ameliorative effect on lipid metabolism, insulin resistance and inflammation in liver as well as in peripheral tissues (95).

In the liver, empagliflozin lowered both hepatic neutral TG and lipotoxic DAG (92, 96–98). These compounds are capable to induce insulin resistance and endoplasmic reticulum stress (99). DAG can activate PKCϵ, which inhibits the insulin receptor by its phosphorylation (98), inducing insulin resistance and endoplasmic reticulum stress (99), both involved in hepatic steatosis development. Empagliflozin administration in the pre-diabetic rats improved hepatic and peripheral insulin resistance mainly decreasing hepatic DAG, but also significantly reducing plasma insulin levels. As a consequence, the rats showed lower blood glucose levels and improved glucose tolerance, decreased fasting glucose, postprandial glucose and AUC, and increased insulin sensitivity in skeletal muscle (95).

In a study conducted on diabetic patients, empagliflozin yielded a reduction of liver fat content (100). Another study investigated the effects of SGLT2-I on markers of oxidative stress, inflammation, liver steatosis and fibrosis in subjects with uncontrolled T2DM and NAFLD. Twenty-six patients treated with metformin were introduced to a SGLT2-I or other glucose-lowering drugs (OTHER) (101). After six months of treatment, a reduction of fatty liver index and the FibroScan result was displayed in the SGLT2-I group. In addition, reduced serum levels of IL-1β, IL-6, TNF, VEGF and MCP-1, and higher levels of IL-4 and IL-10 were reported in the SGLT2-I group. Serum HNE- or MDA-protein adducts (systemic markers of oxidative stress caused by lipid peroxidation) decreased significantly in SGLT2-I patients and correlated with liver steatosis and fibrosis scores. SGLT2-I therapy was associated with reduced liver steatosis and fibrosis markers, circulating pro-inflammatory cytokines and oxidative biomarkers, regardless the glycemic control (101).

#### 4.3.5 Neuroprotective effects

Multiple pathogenic mechanisms have been involved in diabetic peripheral neuropathy (DPN), such as PKC, the polyol pathways, the formation of the advanced glycation end products, and oxidative stress (102). Subsequentially, nerve energy production is reduced (103, 104), accompanied by a disturbance in proteins axonal transport (105). Inhibition of the AMPK pathway hampered mitochondrial function and caused neuronal damage in diabetic rats (106). Conversely, activation of the AMPK expression prevented streptozotocin-induced neuroinflammation enhancing the mitochondrial biogenesis and autophagy in vivo (107), and stimulated the expression of antioxidant enzymes in vitro (108).

In diabetic rats, empagliflozin has been shown to improve sciatic nerve histopathological modifications, scoring, myelination, nerve fibers’ count, and nerve conduction velocity, independently from its anti-hyperglycemic effect. Moreover, it ameliorated motor coordination and mitigated responses to nociceptive stimuli (44). Empagliflozin displayed a protective effect against DPN via regulating the AMPK signaling to reduce oxidative and inflammatory burden, to regulate the extracellular matrix remodeling, and to stimulate autophagy (45, 107). The authors hypothesized a restoration of energy levels, via AMPK activation, as in cardiomyocytes (63, 64). Furthermore, mTor expression resulted reduced by 47% after empagliflozin administration in the sciatic nerve (45). mTOR inhibition might improve DPN through activation of autophagy to remove any damaged cellular constituents, thus increasing the myelin thickness and the myelinated axons (109) and reducing pain (110). Moreover, empagliflozin ameliorated the deleterious effect of inflammatory mediators (IL-1β and TNF-α) by suppressing p-p38 MAPK/p-ERK1/2/p-NF-κB p65 expression (45) (**Figure 2**).

## 5 Discussion

### 5.1 Empagliflozin use in glycogen storage disease type Ib

In GSD type I, the nutritional management is the mainstay of treatment, consisting of high carbohydrate diet, frequent feeding, nocturnal enteral feeding and CS. The aim of nutrition is to achieve a good glycemic and metabolic control and to prevent the long-term complications, such as liver adenoma, hepatocarcinoma, nephropathy and osteoporosis (111). However, patients with GSDIb often present difficulties with the management of nasogastric tube or gastrostomy for the risk of delayed wound healing or infection (6, 23, 26–28, 30, 31), and inflammatory bowel disease.

Data from case reports and a questionnaires from 112 patients showed that treatment with empagliflozin has significant beneficial effects on neutrophil dysfunction, mucosal lesions, recurrent infections, anemia and bowel diseases (6, 18, 23, 24, 26–32). Furthermore, the majority of patients displayed an amelioration of glycemic control with a better fasting tolerance and metabolic parameters (6, 24, 27, 31). Also, increased appetite and recover from tube feeding-dependence has been reported, with an improvement of general well-being (6, 23, 24, 29, 32).

The SGLT2-I empagliflozin, registered for T2DM in adults, has a favorable safety profile. Current evidence from randomized controlled trials in T2DM does not indicate an increased risk of diabetic ketoacidosis and acute kidney injury for SGLT2-I (112). The most common adverse effects are increased urogenital fungal skin infections because of glucosuria. Hypoglycemia has rarely been observed only in association with other antidiabetic therapy (113).

Treating with a glucose-lowering drug patients with a metabolic disease presenting with hypoglycemia might sound counterintuitive, however low rates of hypoglycemia and other side effects, such as urogenital infections, lactic acidosis and ketoacidosis, have been reported so far in the first GSDIb patients treated with empagliflozin (6, 29, 34).

Benefits on glycemic control and fasting tolerance, beyond the favorable effects on neutrophil dysfunction, have been well described in the first study reporting the use of empagliflozin in GSDIb patients at the dose of 0.3-0.7 mg/kg/day (6). Particularly, glycemic profile was assessed by continuous glucose monitoring (CGM) and frequent capillary measurements for a maximum follow-up of 288 days. Mild asymptomatic hypoglycemia was occasionally registered, mostly in the first period of treatment and at a lower frequency than before treatment. Increased appetite was reported after three days in one patient. In another one, continuous enteral feeding was replaced by frequent meals and CS, hypoglycemic episodes quickly became less frequent, and CS was progressively reduced; after one month, the nasogastric tube was dismissed, and fasting intervals increased up three hours. The authors suggested that the improvement of IBD that affects intestinal nutrients absorption might have a positive impact on glycemic and metabolic control (6).

Another report from a GSDIb patient with bowel disease treated with G-CSF and continuous gastric deep feeding showed events of asymptomatic mild hypoglycemia < 3.3 mmol/L (range 2.6–3.3) measured by flash glucose monitoring during the first week of treatment and promptly corrected by oral glucose administration (24). Notably, accuracy of CGM devices in the hypoglycemia range is low and hypoglycemic events should be always verified by glucometer (114–116). However, time below range (TBR) decreased and time in range (TIR) increased also after oral refeeding. An interval of 2.5 h free-gastric drip feeding was introduced in the morning. Asymptomatic mild hypoglycemia was occasionally revealed (2–4 episodes/month) during the next six months by self-monitoring with glucometer (24).

Other case reports displayed an improved glycemic control (27, 31) or did not reveal any increased hypoglycemia (18, 26, 27, 30).

Conversely, in the Halligan paper (29), 4 out 8 patients experienced hypoglycemia after starting empagliflozin. These patients were managed by reducing empagliflozin dose and lightly increasing carbohydrates intake. However, patients were maintained in a tight dietary control in order to minimize carbohydrate intake.

Data from an international retrospective questionnaire study showed hypoglycemia in 20 out 112 patients (18%), lactic acidosis in 6 out 112 (5%). However, hypoglycemia and lactic acidosis are common findings in patients with GSDIb, therefore a contribute of empagliflozin treatment could not be determined. One patient required hospitalizations in intensive care unit for ketoacidosis, during gastroenteritis and dehydration. Therefore, the authors suggested eventually discontinuing empagliflozin during fever or gastrointestinal illnesses for a few days in GSDIb patients, until glucose and fluid homeostasis are stabilized (34). However, in conditions of genetic insulin resistance such as Rabson–Mendenhall syndrome under insulin therapy, empagliflozin improved glycemic control without significantly increasing ketonemia (117, 118).

In the context of metabolic alterations of GSDIb, patients can manifest hyperuricemia and hypertriglyceridemia. Hyperuricemia can result from the inhibitory effect on uric acid tubular excretion by lactate and an increased adenine nucleotides catabolism (119). Although only a few authors reported pre-post SGLT2-I values of uric acid and some cases were concurrently on allopurinol to prevent renal damage (120), empagliflozin seems to have ameliorative effects on hyperuricemia in some GSDIb patients (24, 26, 29, 32), being reported an improvement in the median urate levels (29), with discontinuation of allopurinol in two patients (24, 29). Similar data were evidenced by exploiting untargeted metabolomics profiling in one patient (32). If this may be related to a direct correlation between plasma uric acid and serum 1,5-AG levels, regardless glucosuria, is still unclear (121). Alternatively, this might be reflective of improved metabolic control (29).

TG values were reported to have a variable trend during therapy (26, 27, 29).

Since majority of the studies focused on the effect of empagliflozin on neutrophil dysfunction and inflammatory bowel disease and lack of a long-term follow-up, the current data were scant to evaluate the effect of empagliflozin on the liver, kidney and CV disease in GSDIb patients. However, in two patients with cardiac involvement (heart failure, hypertension and sinus tachycardia), on empagliflozin it was possible to taper the standard cardiological therapy without worsening signs and symptoms (30). This might be related to a possible beneficial effect of empagliflozin on heart function.

In summary, from the existing evidences glycemic and metabolic control appears to be improved in GSDIb patients under treatment with empagliflozin.

### 5.2 Perspectives of empagliflozin use in other metabolic diseases

Data about the systemic effects of empagliflozin have been gathered by trials conducted on big cohorts of T2DM patients to study CV and renal effects (56–58, 75–77), hepatic effects (122), and in preclinical studies (95, 123). Inducing a pseudo-fasting state, empagliflozin stimulates glycogen and lipid catabolism, and ketogenesis, shifting the oxidative metabolism from carbohydrate to lipid utilization. Remarkably, during starvation, cells activate a transcriptional program of adaptation to shortage conditions. The pathways of SIRT1/AMPK and Akt/mTOR act as energy sensors. Empagliflozin can enhance SIRT1/AMPK and inhibit Akt/mTOR activity, leading to decrease lipogenesis, improve mitochondrial function and autophagy, and reduce oxidative stress and inflammation.

Several studies documented that empagliflozin activates the AMPK pathway in cardiomyocytes (65) and in a cardiac ischemia model in mice (64), hearts of lipopolysaccharide-treated mice (63), kidneys of diabetic mice (88), DPN of diabetic rats (45) as well as individual with T2DM (60) and in healthy conditions in vivo and in vitro (63).

Modulation of SIRT1/AMPK and Akt/mTOR pathways is crucial in reducing insulin levels, insulin resistance, glucotoxicity and lipotoxicity, and in increasing ketogenesis (60). At a cardiac level, this translates into reduction of coronary microvascular injury and cardiomyopathy and improvement of contractility (65). In the kidney, it ameliorates glomerular hyperfiltration, tubular damage and inflammation and mitigates the development of nephropathy (124–126). In the liver, empagliflozin protects from lipotoxicity and NAFLD through several mechanisms: reducing lipogenesis, modifying the expression of cytochrome proteins, increasing Nrf2, decreasing fetuin-A and reducing circulating pro-inflammatory cytokines (95). In the peripheral nerve, empagliflozin restores ATP production and enhances autophagy to remove any damaged cellular elements and meet the cellular energy needs (45).

**Figure 3:**
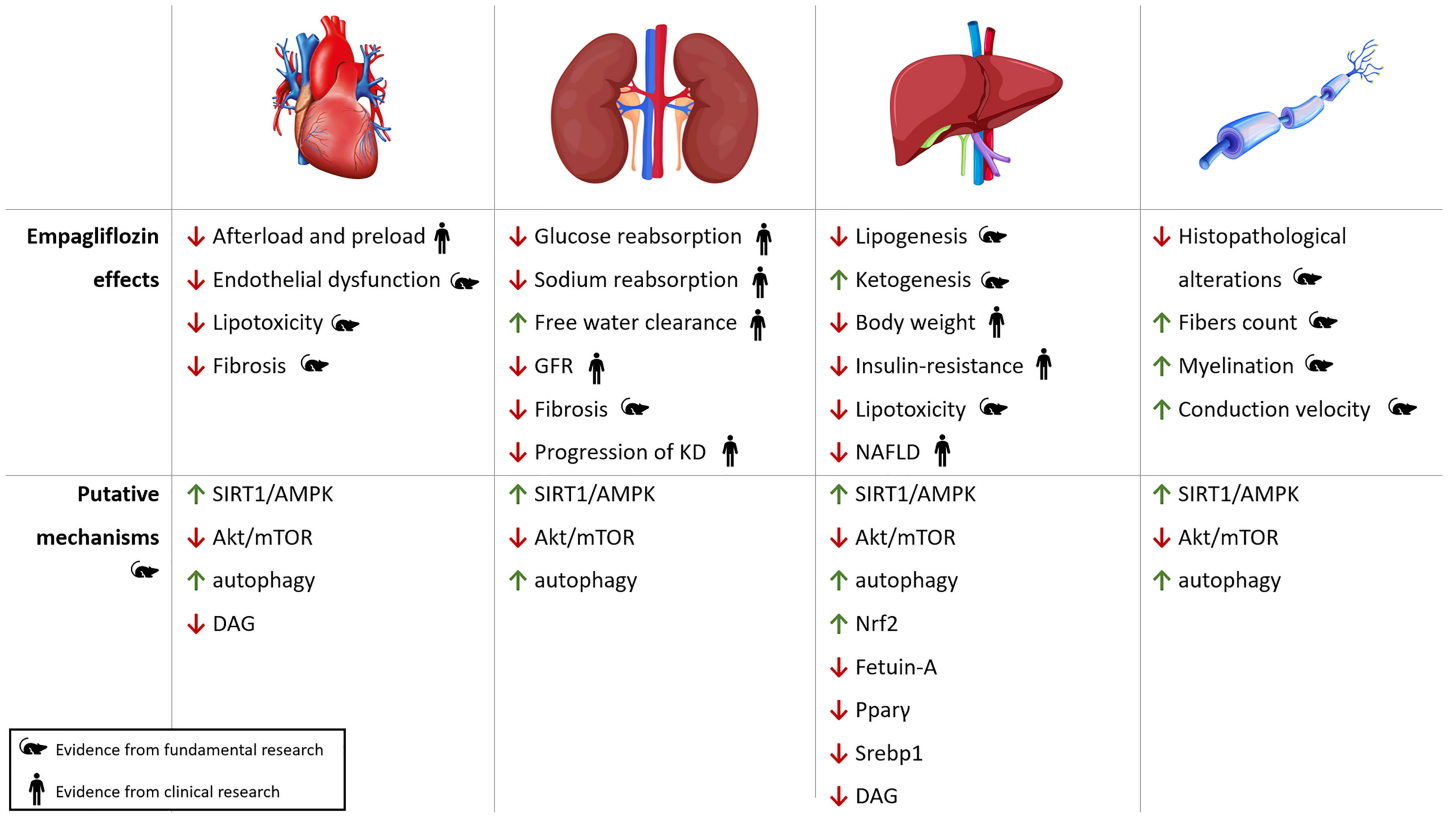
Pleiotropic effects of empagliflozin. Cardiac, renal, hepatic and neuropathic effects of empagliflozin with putative molecular pathways involved in oxidative stress, autophagy and inflammation. KD, kidney disease; NAFLD, Non-Alcoholic Fatty Liver Disease; ROS, reactive oxygen species.

Systemic effects of empagliflozin and its putative molecular mechanisms of action are depicted in **Figure 3**.

The above-mentioned pathways of cellular dysfunction are likely involved in the development of the organ damage in other metabolic diseases presenting with hypoglycemia and/or energy impairment, such as other types of GSDs (3, 127, 128) displaying hepatopathy with several degrees of NAFLD (GSDIa, III, IV, VI, IX, XI), cardiomyopathy (GSDIII), nephropathy (GSDIa); β-oxidation defects (129), mitochondrial disorders (130) with cardiomyopathy, nephropathy, hepatopathy and neuropathy. Furthermore, mitochondrial function and autophagy are defective in organic acidemias (131) and lysosomal storage diseases (132). Therefore, for its multiple effects empagliflozin might be repurposed in other inborn errors of metabolism.

#### 5.2.1 Empagliflozin use in other types of glycogen storage diseases

GSDIa patients present with liver steatosis, because the accumulation of G6P in the endoplasmic reticulum activates lipogenesis (133) and is involved in mitochondrial stress and insulin resistance (134, 135). In GSDIa patients, increased plasma acylcarnitines and abnormal urinary organic acids profile suggested mitochondrial impairment. Remarkably, mitochondrial overload might generate intermediate molecules interfering with the insulin signaling and causing insulin resistance (136). However, insulin resistance and metabolic syndrome can also occur in case of overtreatment in GSDI (137, 138).

Furthermore, the pathogenesis of renal disease in GSDIa and Ib patients is similar to diabetic nephropathy, with initial hyperfiltration evolving towards microalbuminuria, proteinuria, glomerulosclerosis and renal failure (3). A stable renal function was mentioned in a single report of a GSDIb patient treated with empagliflozin (24). Whether empagliflozin use in GSDIb patients might have a protective effect on renal disease needs to be established in prospective trials.

Empagliflozin could be potentially used also in patients with GSDIa with the aim to improve metabolic parameters, mitochondrial stress, insulin resistance, NAFLD and nephropathy.

In GSDIII, the deficiency of the glycogen debranching enzyme results in the accumulation of an abnormal glycogen (limit-dextrin) in liver and muscles. Beyond hypoglycemia and hepatopathy, the cardiac glycogen storage leads to a hypertrophic cardiomyopathy, which can be obstructive and evolve towards heart failure. Standard dietary intervention with high protein and cornstarch supplementation has beneficial effect on glycemic control, but it can worsen glycogen accumulation in the heart. The only effective treatment for cardiomyopathy is ketogenic diet but the outcome strongly depends from patients compliance (127). Empagliflozin might be used to take advantage of its favorable effects on liver and heart.

Dysfunction of autophagy can lead to various liver diseases including NAFLD, steatosis, fibrosis, cirrhosis and hepatocellular carcinoma (139). Liver involvement is a constant finding in hepatic GSDs. Patients with GSDI easily develop steatosis and are at risk for liver adenoma and carcinoma (3, 133). Patients with GSDIII (140), IV, VI, IX, XI are at risk for progressive liver disease and tumor degeneration (127). Mechanisms of inhibition of autophagy are likely to be involved. Remarkably, impaired autophagy has recently been demonstrated in a mouse model of GSDIb (141). For its pro-autophagic effect, empagliflozin may represent a good candidate for the treatment of hepatic GSDs.

Some studies conducted on individuals with muscle GSDs type V and VII reported a beneficial effect of ketogenic diet on mitochondrial function, stimulation of β-oxidation and anaerobic glycolysis, and improvement of the work-out performance (142–145). Since the effect of empagliflozin on oxidative metabolism (shifted from carbohydrate to lipid oxidation) is similar to ketogenic diet, muscle GSDs could benefit of empagliflozin administration in the place or in conjunction with ketogenic diet.

#### 5.2.2 Empagliflozin use in β-oxidation defects, mitochondrial disorders and organic acidemias

Patients with β-oxidation defects present with hypoglycemia, recurrent rhabdomyolysis, hepatopathy, cardiomyopathy and neuropathy. Oxidative disbalance and mitochondrial dysfunction are associated with these disorders (129). Along with patients with mitochondrial disorders and multiorgan involvement, they could benefit of antioxidant and protective effects of empagliflozin on mitochondrial function and mitophagy.

In organic acidemias the accumulation of toxic intermediate metabolism derivatives within mitochondria causes morphological and functional abnormalities leading to severe multiorgan dysfunction. Studies from urinary tubular cells, kidney tubules and explanted livers from methylmalonic acidemia (MMA) patients showed damaged and dysfunctional mitochondria, with generation of ROS and cell distress. A pathway connecting *MMUT* deficiency and mitophagy has recently been identified in mmut-deficient zebrafish. Restoring mitophagy ameliorated disease-relevant phenotypes in mmut-deficient zebrafish. These findings offer potential therapeutic perspectives for repairing mitochondria in MMA (131).

#### 5.2.3 Empagliflozin use in lysosomal storage diseases

Finally, the role of defective autophagy in lysosomal storage diseases (LSDs) is well established. Absent or defective hydrolases or dysregulated endosomal-lysosomal processes lead to accumulation of macromolecules. For some LSDs the enzyme replacement therapy (ERT) is available with variable results. Autophagy inducers have already shown benefit in a few LSDs models (Niemann- Pick type B and C1, Gaucher, Pompe, Ceroid lipofuscinosis neuronal disease type 3). Combination strategies with empagliflozin to induce autophagy might prove more effective than ERT alone (132).

## 6 Conclusion

Although data come from case reports and a retrospective study, the recent off-label use of empagliflozin in GSDIb patients provided evidence of safety and efficacy in improving neutrophil dysfunction and related clinical phenotype. Majority of patients manifested also an improvement in glycemic and metabolic control with a favorable impact on general well-being. Further studies are waited for a more rigorous assessment of the long-term risks and benefits of therapy. Empagliflozin has pleiotropic systemic effects, for which it appears to be a good candidate for drug repurposing also in other metabolic diseases presenting with hypoglycemia, oxidative disbalance and mitochondrial dysfunction, inflammation and defective autophagy, with consequent organ damage.

Future directions through high quality studies should be aimed to provide evidences of safety and efficacy of the empagliflozin use in the inborn errors of metabolism, ranging over amino acids, carbohydrates and fatty acids metabolism defects, mitochondrial and lysosomal diseases.

## Author contributions

AM conceptualized and designed the study, searched for literature, drafted the initial manuscript, and reviewed and approved the final manuscript as submitted. FT and CD-V wrote sections of the manuscript, critically reviewed the manuscript, and approved the final manuscript as submitted. All authors contributed to manuscript revision, read, and approved the submitted version.

## References

[1] Chou JY Jun HS Mansfield BC. Type I glycogen storage diseases: disorders of the glucose-6-phosphatase/glucose-6-phosphate transporter complexes. J Inherit Metab Dis (2015) 38:511–9. doi: 10.1007/s10545-014-9772-x 25288127

[2] Veiga-da-Cunha M Gerin I Chen Y-T de Barsy T de Lonlay P Dionisi-Vici C. A gene on chromosome 11q23 coding for a putative glucose- 6-phosphate translocase is mutated in glycogen-storage disease types ib and ic. Am J Hum Genet (1998) 63:976–83. doi: 10.1086/302068 PMC1377500 9758626

[3] Kishnani PS Austin SL Abdenur JE Arn P Bali DS Boney A. Diagnosis and management of glycogen storage disease type I: a practice guideline of the American college of medical genetics and genomics. Genet Med (2014) 16:e1–e29. doi: 10.1038/gim.2014.128 25356975

[4] Visser G Rake J-P Fernandes J Labrune P Leonard JV Moses S. Neutropenia, neutrophil dysfunction, and inflammatory bowel disease in glycogen storage disease type ib: results of the European study on glycogen storage disease type I. J Pediatr (2000) 137:187–91. doi: 10.1067/mpd.2000.105232 10931410

[5] Chen MA Weinstein DA. Glycogen storage diseases: diagnosis, treatment and outcome. Transl Sci Rare Dis (2016) 1:45–72. doi: 10.3233/TRD-160006

[6] Wortmann SB Van Hove JLK Derks TGJ Chevalier N Knight V Koller A. Treating neutropenia and neutrophil dysfunction in glycogen storage disease type ib with an SGLT2 inhibitor. Blood (2020) 136:1033–43. doi: 10.1182/blood.2019004465 PMC7530374 32294159

[7] Melis D Parenti G Della Casa R Sibilio M Berni Canani R Terrin G. Crohn’s-like ileo-colitis in patients affected by glycogen storage disease ib: two years’ follow-up of patients with a wide spectrum of gastrointestinal signs. Acta Paediatr (2003) 92:1415–21. doi: 10.1080/08035250310007033 14971792

[8] Wicker C Roda C Perry A Arnoux JB Brassier A Castelle M. Infectious and digestive complications in glycogen storage disease type ib: study of a French cohort. Mol Genet Metab Rep (2020) 23:100581. doi: 10.1016/j.ymgmr.2020.100581 32300528 PMC7152669

[9] Rake JP Visser G Labrune P Leonard JV Ullrich K Smit GPA. European Study on glycogen storage disease type I (ESGSD i). guidelines for management of glycogen storage disease type I - European study on glycogen storage disease type I (ESGSD I). Eur J Pediatr (2002) 161 Suppl:S112–9. doi: 10.1007/s00431-002-1016-7 12373584

[10] Visser G Rake J Labrune P Leonard J Moses S Ullrich K. Consensus guidelines for management of glycogen storage disease type 1b - European study on glycogen storage disease type 1. Eur J Pediatr (2002) 161:S120–3. doi: 10.1007/s00431-002-1017-6 12373585

[11] Visser G Rake J Labrune P Leonard J Moses S Ullrich K. Granulocyte colony-stimulating factor in glycogen storage disease type 1b. results of the European study on glycogen storage disease type 1. Eur J Pediatr (2002) 161:S83–7. doi: 10.1007/s00431-002-1010-0 12373578

[12] Li AM Thyagu S Maze D Schreiber R Sirrs S Stockler-Ipsiroglu S. Prolonged granulocyte colony stimulating factor use in glycogen storage disease type 1b associated with acute myeloid leukemia and with shortened telomere length. Pediatr Hematol Oncol (2018) 35:45–51. doi: 10.1080/08880018.2018.1440675 29652549

[13] Dale DC Bolyard AA Marrero T Kelley ML Makaryan V Tran E. Neutropenia in glycogen storage disease ib. Curr Opin Hematol (2019) 26:16–21. doi: 10.1097/MOH.0000000000000474 30451720 PMC7000169

[14] Veiga-da-Cunha M Chevalier N Stephenne X Defour JP Paczia N Ferster A. Failure to eliminate a phosphorylated glucose analog leads to neutropenia in patients with G6PT and G6PC3 deficiency. Proc Natl Acad Sci USA (2019) 116:1241–50. doi: 10.1073/pnas.1816143116 PMC6347702 30626647

[15] Yamanouchi T Tachibana Y Akanuma H Minoda S Shinohara T Moromizato H. Origin and disposal of 1,5-anhydroglucitol, a major polyol in the human body. Am J Physiol Metab (1992) 263:E268–73. doi: 10.1152/ajpendo.1992.263.2.E268 1514606

[16] Crane RK Sols A. The non-competitive inhibition of brain hexokinase by glucose-6-Phosphate and related compounds. J Biol Chem (1954) 210:597–606. doi: 10.1016/S0021-9258(18)65385-2 13211596

[17] Boulanger C Stephenne X Diederich J Mounkoro P Chevalier N Ferster A. Successful use of empagliflozin to treat neutropenia in two G6PC3-deficient children: impact of a mutation in SGLT5. J Inherit Metab Dis (2022) 45:759–68. doi: 10.1002/jimd.12509 PMC9540799 35506446

[18] Makrilakis K Barmpagianni A Veiga-da-Cunha M. Repurposing of empagliflozin as a possible treatment for neutropenia and inflammatory bowel disease in glycogen storage disease type ib: a case report. Cureus (2022) 14:12–5. doi: 10.7759/cureus.27264 PMC9403211 36039216

[19] Akanuma Y Morita M Fukuzawa N Yamanouchi T Akanuma H. Urinary excretion of 1,5-anhydro-D-glucitol accompanying glucose excretion in diabetic patients. Diabetologia (1988) 31:831–5. doi: 10.1007/BF00277486 3234638

[20] Chao EC Henry RR. SGLT2 inhibition — a novel strategy for diabetes treatment. Nat Rev Drug Discov (2010) 9:551–9. doi: 10.1038/nrd3180 20508640

[21] Fortuna D McCloskey LJ Stickle DF. Model analysis of effect of canagliflozin (Invokana), a sodium–glucose cotransporter 2 inhibitor, to alter plasma 1,5-anhydroglucitol. Clin Chim Acta (2016) 452:138–41. doi: 10.1016/j.cca.2015.11.010 26569347

[22] DeFronzo RA Hompesch M Kasichayanula S Liu X Hong Y Pfister M. Characterization of renal glucose reabsorption in response to dapagliflozin in healthy subjects and subjects with type 2 diabetes. Diabetes Care (2013) 36:3169–76. doi: 10.2337/dc13-0387 PMC3781504 23735727

[23] Grünert SC Elling R Maag B Wortmann SB Derks TGJ Hannibal L. Improved inflammatory bowel disease, wound healing and normal oxidative burst under treatment with empagliflozin in glycogen storage disease type ib. Orphanet J Rare Dis (2020) 15:218. doi: 10.1186/s13023-020-01503-8 32838757 PMC7446198

[24] Rossi A Miele E Fecarotta S Veiga-da-Cunha M Martinelli M Mollica C. Crohn disease-like enterocolitis remission after empagliflozin treatment in a child with glycogen storage disease type ib: a case report. Ital J Pediatr (2021) 47:149. doi: 10.1186/s13052-021-01100-w 34215305 PMC8254289

[25] Ceccarani C Bassanini G Montanari C Casiraghi MC Ottaviano E Morace G. Proteobacteria overgrowth and butyrate-producing taxa depletion in the gut microbiota of glycogen storage disease type 1 patients. Metabolites (2020) 10:133. doi: 10.3390/metabo10040133 32235604 PMC7240959

[26] Mikami M Arai A Mizumoto H. Empagliflozin ameliorated neutropenia in a girl with glycogen storage disease ib. Pediatr Int (2021) 63:1394–6. doi: 10.1111/ped.14629 34378838

[27] Kaczor M Greczan M Kierus K Ehmke vel Emczyńska-Seliga E Ciara E Piątosa B. Sodium-glucose cotransporter type 2 channel inhibitor: breakthrough in the treatment of neutropenia in patients with glycogen storage disease type 1b? JIMD Rep (2022) 63:199–206. doi: 10.1002/jmd2.12278 35433171 PMC8995836

[28] Grünert SC Rosenbaum-Fabian S Schumann A Selbitz A Merz W Gieselmann A. Two successful pregnancies and first use of empagliflozin during pregnancy in glycogen storage disease type ib. JIMD Rep (2022) 63:303–8. doi: 10.1002/jmd2.12295 PMC9259388 35822091

[29] Halligan RK Dalton RN Turner C Lewis KA Mundy HR. Understanding the role of SGLT2 inhibitors in glycogen storage disease type ib: the experience of one UK centre. Orphanet J Rare Dis (2022) 17:195. doi: 10.1186/s13023-022-02345-2 35549996 PMC9096769

[30] Bidiuk J Gaciong Z Sobieraj P. The overall benefits of empagliflozin treatment in adult siblings with glycogen storage disease type ib: one year experience. Arch Med Sci (2022) 18:1095–9. doi: 10.5114/aoms/150029 PMC9266796 35982912

[31] Hexner-Erlichman Z Veiga-da-Cunha M Zehavi Y Vadasz Z Sabag AD Tatour S. Favorable outcome of empagliflozin treatment in two pediatric glycogen storage disease type 1b patients. Front Pediatr (2022) 10:1071464. doi: 10.3389/fped.2022.1071464 36507137 PMC9727171

[32] Tallis E Karsenty CL Grimes AB Karam LB Elsea SH Sutton VR. Untargeted metabolomic profiling in a patient with glycogen storage disease ib receiving empagliflozin treatment. JIMD Rep (2022) 63:309–15. doi: 10.1002/jmd2.12304 PMC9259396 35822097

[33] Mathis T Poms M Köfeler H Gautschi M Plecko B Baumgartner MR. Untargeted plasma metabolomics identifies broad metabolic perturbations in glycogen storage disease type I. J Inherit Metab Dis (2022) 45:235–47. doi: 10.1002/jimd.12451 PMC9299190 34671989

[34] Grünert SC Derks TGJ Adrian K Al-Thihli K Ballhausen D Bidiuk J. Efficacy and safety of empagliflozin in glycogen storage disease type ib: data from an international questionnaire. Genet Med (2022) 24:1781–8. doi: 10.1016/j.gim.2022.04.001 35503103

[35] Hiwarkar P Bargir U Pandrowala A Bodhanwala M Thakker N Taur P. SLGT2 inhibitor rescues myelopoiesis in G6PC3 deficiency. J Clin Immunol (2022) 42:1653–9. doi: 10.1007/s10875-022-01323-4 35838821

[36] Yu S. The anhydrofructose pathway of glycogen catabolism. IUBMB Life (2008) 60:798–809. doi: 10.1002/iub.125 18785261

[37] Ferrannini E Natali A Camastra S Nannipieri M Mari A Adam K-P. Early metabolic markers of the development of dysglycemia and type 2 diabetes and their physiological significance. Diabetes (2013) 62:1730–7. doi: 10.2337/db12-0707 PMC3636608 23160532

[38] Chen LH Leung PS. Inhibition of the sodium glucose co-transporter-2: its beneficial action and potential combination therapy for type 2 diabetes mellitus. Diabetes Obes Metab (2013) 15:392–402. doi: 10.1111/dom.12064 23331516

[39] Heerspink HJL Perkins BA Fitchett DH Husain M Cherney DZI. Sodium glucose cotransporter 2 inhibitors in the treatment of diabetes mellitus. Circulation (2016) 134:752–72. doi: 10.1161/CIRCULATIONAHA.116.021887 27470878

[40] Chatterjee S Khunti K Davies MJ. Optimizing management of glycaemia. Best Pract Res Clin Endocrinol Metab (2016) 30:397–411. doi: 10.1016/j.beem.2016.06.002 27432074

[41] Michel MC Mayoux E Vallon V. A comprehensive review of the pharmacodynamics of the SGLT2 inhibitor empagliflozin in animals and humans. Naunyn Schmiedebergs Arch Pharmacol (2015) 388:801–16. doi: 10.1007/s00210-015-1134-1 PMC5896322 26108304

[42] Forycka J Hajdys J Krzemińska J Wilczopolski P Wronka M Młynarska E. New insights into the use of empagliflozin–a comprehensive review. Biomedicines (2022) 10:3294. doi: 10.3390/biomedicines10123294 36552050 PMC9775057

[43] Nakamura K Miyoshi T Yoshida M Akagi S Saito Y Ejiri K. Pathophysiology and treatment of diabetic cardiomyopathy and heart failure in patients with diabetes mellitus. Int J Mol Sci (2022) 23:3587. doi: 10.3390/ijms23073587 35408946 PMC8999085

[44] Andreadi A Bellia A Di Daniele N Meloni M Lauro R Della-Morte D. The molecular link between oxidative stress, insulin resistance, and type 2 diabetes: a target for new therapies against cardiovascular diseases. Curr Opin Pharmacol (2022) 62:85–96. doi: 10.1016/j.coph.2021.11.010 34959126

[45] Abdelkader NF Elbaset MA Moustafa PE Ibrahim SM. Empagliflozin mitigates type 2 diabetes-associated peripheral neuropathy: a glucose-independent effect through AMPK signaling. Arch Pharm Res (2022) 45:475–93. doi: 10.1007/s12272-022-01391-5 PMC9325846 35767208

[46] Shimabukuro M. Cardiac adiposity and global cardiometabolic risk new concept and clinical implication. Circ J (2009) 73:27–34. doi: 10.1253/circj.CJ-08-1012 19057089

[47] Maiorana A Del Bianco C Cianfarani S. Adipose tissue: a metabolic regulator. potential implications for the metabolic outcome of subjects born small for gestational age (SGA). Rev Diabetes Stud (2007) 4:134–46. doi: 10.1900/RDS.2007.4.134 PMC2174062 18084671

[48] Nakamura K Miyoshi T Yunoki K Ito H. Postprandial hyperlipidemia as a potential residual risk factor. J Cardiol (2016) 67:335–9. doi: 10.1016/j.jjcc.2015.12.001 26744235

[49] Yoshida M Nakamura K Miyoshi T Yoshida M Kondo M Akazawa K. Combination therapy with pemafibrate (K-877) and pitavastatin improves vascular endothelial dysfunction in dahl/salt-sensitive rats fed a high-salt and high-fat diet. Cardiovasc Diabetol (2020) 19:149. doi: 10.1186/s12933-020-01132-2 32979918 PMC7520032

[50] Inzucchi SE Zinman B Wanner C Ferrari R Fitchett D Hantel S. SGLT-2 inhibitors and cardiovascular risk: proposed pathways and review of ongoing outcome trials. Diabetes Vasc Dis Res (2015) 12:90–100. doi: 10.1177/1479164114559852 PMC4361459 25589482

[51] Spiga R Marini MA Mancuso E Di Fatta C Fuoco A Perticone F. Uric acid is associated with inflammatory biomarkers and induces inflammation Via activating the NF-κB signaling pathway in HepG2 cells. Arterioscler Thromb Vasc Biol (2017) 37:1241–9. doi: 10.1161/ATVBAHA.117.309128 28408375

[52] La Grotta R de Candia P Olivieri F Matacchione G Giuliani A Rippo MR. Anti-inflammatory effect of SGLT-2 inhibitors via uric acid and insulin. Cell Mol Life Sci (2022) 79:273. doi: 10.1007/s00018-022-04289-z 35503137 PMC9064844

[53] Dong M Chen H Wen S Yuan Y Yang L Xu D. The mechanism of sodium-glucose cotransporter-2 inhibitors in reducing uric acid in type 2 diabetes mellitus. Diabetes Metab Syndr Obes (2023) 16:437–45. doi: 10.2147/DMSO.S399343 PMC9938669 36820272

[54] Mauer J Chaurasia B Plum L Quast T Hampel B Blüher M. Myeloid cell-restricted insulin receptor deficiency protects against obesity-induced inflammation and systemic insulin resistance. PloS Genet (2010) 6:e1000938. doi: 10.1371/journal.pgen.1000938 20463885 PMC2865520

[55] Aljada A Ghanim H Saadeh R Dandona P. Insulin inhibits NFκB and MCP-1 expression in human aortic endothelial cells. J Clin Endocrinol Metab (2001) 86:450–3. doi: 10.1210/jcem.86.1.7278 11232040

[56] Borghi C Bragagni A. The new type 2 diabetes mellitus therapy: comparison between the two classes of drugs GLPR (glucagon-like peptide receptor) agonists and SGLT2 (sodium–glucose cotransporter 2) inhibitors. Eur Hear J Suppl (2020) 22:L28–32. doi: 10.1093/eurheartj/suaa129 PMC7673624 33239977

[57] Packer M Anker SD Butler J Filippatos G Pocock SJ Carson P. Cardiovascular and renal outcomes with empagliflozin in heart failure. N Engl J Med (2020) 383:1413–24. doi: 10.1056/NEJMoa2022190 32865377

[58] Williams DM Evans M. Are SGLT-2 inhibitors the future of heart failure treatment? the EMPEROR-preserved and EMPEROR-reduced trials. Diabetes Ther (2020) 11:1925–34. doi: 10.1007/s13300-020-00889-9 PMC7434820 32710261

[59] Santer R Calado J. Familial renal glucosuria and SGLT2: from a mendelian trait to a therapeutic target. Clin J Am Soc Nephrol (2010) 5:133–41. doi: 10.2215/CJN.04010609 19965550

[60] Packer M. SGLT2 inhibitors produce cardiorenal benefits by promoting adaptive cellular reprogramming to induce a state of fasting mimicry: a paradigm shift in understanding their mechanism of action. Diabetes Care (2020) 43:508–11. doi: 10.2337/dci19-0074 32079684

[61] Nakamura M Sadoshima J. Cardiomyopathy in obesity, insulin resistance and diabetes. J Physiol (2020) 598:2977–93. doi: 10.1113/JP276747 30869158

[62] Volpe CMO Villar-Delfino PH dos Anjos PMF Nogueira-Machado JA. Cellular death, reactive oxygen species (ROS) and diabetic complications. Cell Death Dis (2018) 9:119. doi: 10.1038/s41419-017-0135-z 29371661 PMC5833737

[63] Koyani CN Plastira I Sourij H Hallström S Schmidt A Rainer PP. Empagliflozin protects heart from inflammation and energy depletion via AMPK activation. Pharmacol Res (2020) 158:104870. doi: 10.1016/j.phrs.2020.104870 32434052

[64] Lu Q Liu J Li X Sun X Zhang J Ren D. Empagliflozin attenuates ischemia and reperfusion injury through LKB1/AMPK signaling pathway. Mol Cell Endocrinol (2020) 501:110642. doi: 10.1016/j.mce.2019.110642 31759100

[65] Zhou H Wang S Zhu P Hu S Chen Y Ren J. Empagliflozin rescues diabetic myocardial microvascular injury via AMPK-mediated inhibition of mitochondrial fission. Redox Biol (2018) 15:335–46. doi: 10.1016/j.redox.2017.12.019 PMC5756062 29306791

[66] Zheng H Zhu H Liu X Huang X Huang A Huang Y. Mitophagy in diabetic cardiomyopathy: roles and mechanisms. Front Cell Dev Biol (2021) 9:750382. doi: 10.3389/fcell.2021.750382 34646830 PMC8503602

[67] Kato ET Kimura T. Sodium-glucose Co-transporters-2 inhibitors and heart failure: state of the art review and future potentials. Int J Hear Fail (2020) 2:12. doi: 10.36628/ijhf.2019.0013 PMC9536731 36263075

[68] Zelniker TA Wiviott SD Raz I Im K Goodrich EL Bonaca MP. SGLT2 inhibitors for primary and secondary prevention of cardiovascular and renal outcomes in type 2 diabetes: a systematic review and meta-analysis of cardiovascular outcome trials. Lancet (2019) 393:31–9. doi: 10.1016/S0140-6736(18)32590-X 30424892

[69] Das NA Carpenter AJ Belenchia A Aroor AR Noda M Siebenlist U. Empagliflozin reduces high glucose-induced oxidative stress and miR-21-dependent TRAF3IP2 induction and RECK suppression, and inhibits human renal proximal tubular epithelial cell migration and epithelial-to-mesenchymal transition. Cell Signal (2020) 68:109506. doi: 10.1016/j.cellsig.2019.109506 31862399 PMC7493965

[70] Quagliariello V De Laurentiis M Rea D Barbieri A Monti MG Carbone A. The SGLT-2 inhibitor empagliflozin improves myocardial strain, reduces cardiac fibrosis and pro-inflammatory cytokines in non-diabetic mice treated with doxorubicin. Cardiovasc Diabetol (2021) 20:150. doi: 10.1186/s12933-021-01346-y 34301253 PMC8305868

[71] Zeng S Delic D Chu C Xiong Y Luo T Chen X. Antifibrotic effects of low dose SGLT2 inhibition with empagliflozin in comparison to ang II receptor blockade with telmisartan in 5/6 nephrectomised rats on high salt diet. BioMed Pharmacother (2022) 146:112606. doi: 10.1016/j.biopha.2021.112606 34968924

[72] Santos-Ferreira D Gonçalves-Teixeira P Fontes-Carvalho R. SGLT-2 inhibitors in heart failure and type-2 diabetes: hitting two birds with one stone? Cardiology (2020) 145:311–20. doi: 10.1159/000504694 31865310

[73] Cherney DZI Perkins BA Soleymanlou N Maione M Lai V Lee A. Renal hemodynamic effect of sodium-glucose cotransporter 2 inhibition in patients with type 1 diabetes mellitus. Circulation (2014) 129:587–97. doi: 10.1161/CIRCULATIONAHA.113.005081 24334175

[74] Zelniker TA Braunwald E. Cardiac and renal effects of sodium-glucose Co-transporter 2 inhibitors in diabetes: JACC state-of-the-Art review. J Am Coll Cardiol (2018) 72:1845–55. doi: 10.1016/j.jacc.2018.06.040 30075873

[75] Zinman B Wanner C Lachin JM Fitchett D Bluhmki E Hantel S. Empagliflozin, cardiovascular outcomes, and mortality in type 2 diabetes. N Engl J Med (2015) 373:2117–28. doi: 10.1056/NEJMoa1504720 26378978

[76] Packer M Butler J Zannad F Filippatos G Ferreira JP Pocock SJ. Effect of empagliflozin on worsening heart failure events in patients with heart failure and preserved ejection fraction: EMPEROR-preserved trial. Circulation (2021) 144:1284–94. doi: 10.1161/CIRCULATIONAHA.121.056824 PMC8522627 34459213

[77] Packer M Butler J Zannad F Pocock SJ Filippatos G Ferreira JP. Empagliflozin and major renal outcomes in heart failure. N Engl J Med (2021) 385:1531–3. doi: 10.1056/NEJMc2112411 34449179

[78] Wanner C. EMPA-REG OUTCOME: the nephrologist’s point of view. Am J Cardiol (2017) 120:S59–67. doi: 10.1016/j.amjcard.2017.05.012 28606346

[79] Kelly MS Lewis J Huntsberry AM Dea L Portillo I. Efficacy and renal outcomes of SGLT2 inhibitors in patients with type 2 diabetes and chronic kidney disease. Postgrad Med (2019) 131:31–42. doi: 10.1080/00325481.2019.1549459 30449220

[80] Braunwald E. SGLT2 inhibitors: the statins of the 21stcentury. Eur Heart J (2022) 43:1029–30. doi: 10.1093/eurheartj/ehab765 34741610

[81] Sizar O Podder V Talati R. Empagliflozin. 2215 Constitution Avenue, N.W. Washington, DC: The American Pharmacists Association (2022) p. 45–47 p. doi: 10.21019/druginformation.empagliflozin

[82] Cherney D Lund SS Perkins BA Groop P-H Cooper ME Kaspers S. The effect of sodium glucose cotransporter 2 inhibition with empagliflozin on microalbuminuria and macroalbuminuria in patients with type 2 diabetes. Diabetologia (2016) 59:1860–70. doi: 10.1007/s00125-016-4008-2 27316632

[83] American Diabetes Association. 10. cardiovascular disease and risk management: standards of medical care in diabetes–2021. Diabetes Care (2021) 44:S125–50. doi: 10.2337/dc21-S010 33298421

[84] Szrejder M Piwkowska A. AMPK signalling: implications for podocyte biology in diabetic nephropathy. Biol Cell (2019) 111:109–20. doi: 10.1111/boc.201800077 30702162

[85] Kitada M Kume S Takeda-Watanabe A Kanasaki K Koya D. Sirtuins and renal diseases: relationship with aging and diabetic nephropathy. Clin Sci (2013) 124:153–64. doi: 10.1042/CS20120190 PMC3466784 23075334

[86] Takahashi A Takabatake Y Kimura T Maejima I Namba T Yamamoto T. Autophagy inhibits the accumulation of advanced glycation end products by promoting lysosomal biogenesis and function in the kidney proximal tubules. Diabetes (2017) 66:1359–72. doi: 10.2337/db16-0397 28246295

[87] Umino H Hasegawa K Minakuchi H Muraoka H Kawaguchi T Kanda T. High basolateral glucose increases sodium-glucose cotransporter 2 and reduces sirtuin-1 in renal tubules through glucose transporter-2 detection. Sci Rep (2018) 8:6791. doi: 10.1038/s41598-018-25054-y 29717156 PMC5931531

[88] Lee YH Kim SH Kang JM Heo JH Kim D-J Park SH. Empagliflozin attenuates diabetic tubulopathy by improving mitochondrial fragmentation and autophagy. Am J Physiol Physiol (2019) 317:F767–80. doi: 10.1152/ajprenal.00565.2018 31390268

[89] Hardy T Oakley F Anstee QM Day CP. Nonalcoholic fatty liver disease: pathogenesis and disease spectrum. Annu Rev Pathol Mech Dis (2016) 11:451–96. doi: 10.1146/annurev-pathol-012615-044224 26980160

[90] Younossi Z Anstee QM Marietti M Hardy T Henry L Eslam M. Global burden of NAFLD and NASH: trends, predictions, risk factors and prevention. Nat Rev Gastroenterol Hepatol (2018) 15:11–20. doi: 10.1038/nrgastro.2017.109 28930295

[91] Caussy C Aubin A Loomba R. The relationship between type 2 diabetes, NAFLD, and cardiovascular risk. Curr Diabetes Rep (2021) 21:15. doi: 10.1007/s11892-021-01383-7 PMC8805985 33742318

[92] Dogru T Kirik A Gurel H Rizvi AA Rizzo M Sonmez A. The evolving role of fetuin-a in nonalcoholic fatty liver disease: an overview from liver to the heart. Int J Mol Sci (2021) 22:6627. doi: 10.3390/ijms22126627 34205674 PMC8234007

[93] Mori K Emoto M Inaba M. Fetuin-a: a multifunctional protein. Recent Pat Endocr Metab Immune Drug Discov (2011) 5:124–46. doi: 10.2174/187221411799015372 22074587

[94] Lee K-Y Lee W Jung S-H Park J Sim H Choi Y-J. Hepatic upregulation of fetuin-a mediates acetaminophen-induced liver injury through activation of TLR4 in mice. Biochem Pharmacol (2019) 166:46–55. doi: 10.1016/j.bcp.2019.05.011 31077645

[95] Hüttl M Markova I Miklankova D Zapletalova I Poruba M Haluzik M. In a prediabetic model, empagliflozin improves hepatic lipid metabolism independently of obesity and before onset of hyperglycemia. Int J Mol Sci (2021) 22:11513. doi: 10.3390/ijms222111513 34768942 PMC8584090

[96] Ashino T Ohkubo-Morita H Yamamoto M Yoshida T Numazawa S. Possible involvement of nuclear factor erythroid 2-related factor 2 in the gene expression of Cyp2b10 and Cyp2a5. Redox Biol (2014) 2:284–8. doi: 10.1016/j.redox.2013.12.025 PMC3909825 24494203

[97] Guerra S Gastaldelli A. The role of the liver in the modulation of glucose and insulin in non alcoholic fatty liver disease and type 2 diabetes. Curr Opin Pharmacol (2020) 55:165–74. doi: 10.1016/j.coph.2020.10.016 33278735

[98] Petersen MC Shulman GI. Roles of diacylglycerols and ceramides in hepatic insulin resistance. Trends Pharmacol Sci (2017) 38:649–65. doi: 10.1016/j.tips.2017.04.004 PMC5499157 28551355

[99] Lebeaupin C Vallée D Hazari Y Hetz C Chevet E Bailly-Maitre B. Endoplasmic reticulum stress signalling and the pathogenesis of non-alcoholic fatty liver disease. J Hepatol (2018) 69:927–47. doi: 10.1016/j.jhep.2018.06.008 29940269

[100] Pokharel A KCS Thapa P Karki N Shrestha R Jaishi B Paudel MS. The effect of empagliflozin on liver fat in type 2 diabetes mellitus patients with non-alcoholic fatty liver disease. Cureus (2021) 2. doi: 10.7759/cureus.16687 PMC8394637 34466320

[101] Bellanti F Lo BA Dobrakowski M Kasperczyk A Kasperczyk S Aich P. Impact of sodium glucose cotransporter-2 inhibitors on liver steatosis/fibrosis/inflammation and redox balance in non-alcoholic fatty liver disease. World J Gastroenterol (2022) 28:3243–57. doi: 10.3748/wjg.v28.i26.3243 PMC9331534 36051336

[102] Duby JJ Campbell RK Setter SM White JR Rasmussen KA. Diabetic neuropathy: an intensive review. Am J Heal Pharm (2004) 61:160–73. doi: 10.1093/ajhp/61.2.160 14750401

[103] Obrosova IG. How does glucose generate oxidative stress in peripheral nerve? Int Rev Neurobiol (2002), 3–35. doi: 10.1016/S0074-7742(02)50071-4 12198815

[104] Vincent AM Russell JW Low P Feldman EL. Oxidative stress in the pathogenesis of diabetic neuropathy. Endocr Rev (2004) 25:612–28. doi: 10.1210/er.2003-0019 15294884

[105] Fernyhough P Schmidt RE. Neurofilaments in diabetic neuropathy. Int Rev Neurobiol (2002), 115–44. doi: 10.1016/S0074-7742(02)50075-1 12198808

[106] Roy Chowdhury SK Smith DR Saleh A Schapansky J Marquez A Gomes S. Impaired adenosine monophosphate-activated protein kinase signalling in dorsal root ganglia neurons is linked to mitochondrial dysfunction and peripheral neuropathy in diabetes. Brain (2012) 135:1751–66. doi: 10.1093/brain/aws097 PMC3359752 22561641

[107] Yerra VG Kumar A. Adenosine monophosphate-activated protein kinase abates hyperglycaemia-induced neuronal injury in experimental models of diabetic neuropathy: effects on mitochondrial biogenesis, autophagy and neuroinflammation. Mol Neurobiol (2017) 54:2301–12. doi: 10.1007/s12035-016-9824-3 26957299

[108] Lin C-H Cheng Y-C Nicol CJ Lin K-H Yen C-H Chiang M-C. Activation of AMPK is neuroprotective in the oxidative stress by advanced glycosylation end products in human neural stem cells. Exp Cell Res (2017) 359:367–73. doi: 10.1016/j.yexcr.2017.08.019 28821394

[109] Liu S-Y Chen L Li X-C Hu Q-K He L-J. Lycium barbarum polysaccharide protects diabetic peripheral neuropathy by enhancing autophagy via mTOR/p70S6K inhibition in streptozotocin-induced diabetic rats. J Chem Neuroanat (2018) 89:37–42. doi: 10.1016/j.jchemneu.2017.12.011 29294366

[110] Russell RC Tian Y Yuan H Park HW Chang Y-Y Kim J. ULK1 induces autophagy by phosphorylating beclin-1 and activating VPS34 lipid kinase. Nat Cell Biol (2013) 15:741–50. doi: 10.1038/ncb2757 PMC3885611 23685627

[111] Steunenberg TAH Peeks F Hoogeveen IJ Mitchell JJ Mundy H de Boer F. Safety issues associated with dietary management in patients with hepatic glycogen storage disease. Mol Genet Metab (2018) 125:79–85. doi: 10.1016/j.ymgme.2018.07.004 30037503

[112] Donnan JR Grandy CA Chibrikov E Marra CA Aubrey-Bassler K Johnston K. Comparative safety of the sodium glucose co-transporter 2 (SGLT2) inhibitors: a systematic review and meta-analysis. BMJ Open (2019) 9:1–15. doi: 10.1136/bmjopen-2018-022577 PMC6361337 30813108

[113] Rosenstock J Jelaska A Frappin G Salsali A Kim G Woerle HJ. Improved glucose control with weight loss, lower insulin doses, and no increased hypoglycemia with empagliflozin added to titrated multiple daily injections of insulin in obese inadequately controlled type 2 diabetes. Diabetes Care (2014) 37:1815–23. doi: 10.2337/dc13-3055 24929430

[114] Worth C Hoskyns L Salomon-Estebanez M Nutter PW Harper S Derks TG. Continuous glucose monitoring for children with hypoglycaemia: evidence in 2023. Front Endocrinol (Lausanne) (2023) 14:1116864. doi: 10.3389/fendo.2023.1116864 36755920 PMC9900115

[115] Rayannavar A Elci OU Mitteer L De León DD. Continuous glucose monitoring systems: are they useful for evaluating glycemic control in children with hyperinsulinism? Horm Res Paediatr (2019) 92:319–27. doi: 10.1159/000506230 PMC7192768 32208390

[116] Alsaffar H Turner L Yung Z O’Hara C Didi M Senniappan S. Flash glucose monitoring in children with congenital hyperinsulinism; first report on accuracy and patient experience. Endocr Abstr (2016), 4–9. doi: 10.1530/endoabs.45.P55 PMC5870486 29599801

[117] dos Santos SS Ramaldes LA Gabbay MAL Moises RCS Dib SA. Use of a sodium-glucose cotransporter 2 inhibitor, empagliflozin, in a patient with rabson-mendenhall syndrome. Horm Res Paediatr (2021) 94:313–6. doi: 10.1159/000519613 34551418

[118] Galderisi A Tamborlane W Taylor SI Attia N Moretti C Barbetti F. SGLT2i improves glycemic control in patients with congenital severe insulin resistance. Pediatrics (2022) 150:1–4. doi: 10.1542/peds.2021-055671 35652305

[119] Chen Y-T Kishnani PS Koeberl D. Glycogen storage diseases. In: Valle DL Antonarakis S Ballabio A Beaudet AL Mitchell GA, editors. The online metabolic and molecular bases of inherited disease. New York, NY: McGraw-Hill Education (2019). Available at: http://ommbid.mhmedical.com/content.aspx?aid=1181420647.

[120] Ng ES-T Gupta S Khin SM Mak A. Gout, anemia, and hepatomegaly in a young man with glycogen storage disease. JCR J Clin Rheumatol (2012) 18:222–3. doi: 10.1097/RHU.0b013e3182598ed1 22653625

[121] Ouchi M Oba K Aoyama J Watanabe K Ishii K Yano H. Serum uric acid in relation to serum 1,5-anhydroglucitol levels in patients with and without type 2 diabetes mellitus. Clin Biochem (2013) 46:1436–41. doi: 10.1016/j.clinbiochem.2013.06.003 23778057

[122] Dougherty JA Guirguis E Thornby K-A. A systematic review of newer antidiabetic agents in the treatment of nonalcoholic fatty liver disease. Ann Pharmacother (2021) 55:65–79. doi: 10.1177/1060028020935105 32571083

[123] Han JH Oh TJ Lee G Maeng HJ Lee DH Kim KM. The beneficial effects of empagliflozin, an SGLT2 inhibitor, on atherosclerosis in ApoE –/– mice fed a western diet. Diabetologia (2017) 60:364–76. doi: 10.1007/s00125-016-4158-2 27866224

[124] Terami N Ogawa D Tachibana H Hatanaka T Wada J Nakatsuka A. Long-term treatment with the sodium glucose cotransporter 2 inhibitor, dapagliflozin, ameliorates glucose homeostasis and diabetic nephropathy in db/db mice. PloS One (2014) 9:e100777. doi: 10.1371/journal.pone.0100777 24960177 PMC4069074

[125] Udwan K Abed A Roth I Dizin E Maillard M Bettoni C. Dietary sodium induces a redistribution of the tubular metabolic workload. J Physiol (2017) 595:6905–22. doi: 10.1113/JP274927 PMC5685825 28940314

[126] Huang DY Gao H Boini KM Osswald H Nürnberg B Lang F. In vivo Stimulation of AMP-activated protein kinase enhanced tubuloglomerular feedback but reduced tubular sodium transport during high dietary NaCl intake. Pflügers Arch - Eur J Physiol (2010) 460:187–96. doi: 10.1007/s00424-010-0803-7 20349193

[127] Rossi A Hoogeveen IJ Bastek VB Boer F Montanari C Meyer U. Dietary lipids in glycogen storage disease type III: a systematic literature study, case studies, and future recommendations. J Inherit Metab Dis (2020) 43:770–7. doi: 10.1002/jimd.12224 PMC7383479 32064649

[128] Massese M Tagliaferri F Dionisi-Vici C Maiorana A. Glycogen storage diseases with liver involvement: a literature review of GSD type 0, IV, VI, IX and XI. Orphanet J Rare Dis (2022) 17:1–12. doi: 10.1186/s13023-022-02387-6 35725468 PMC9208159

[129] Ribas GS Vargas CR. Evidence that oxidative disbalance and mitochondrial dysfunction are involved in the pathophysiology of fatty acid oxidation disorders. Cell Mol Neurobiol (2022) 42:521–32. doi: 10.1007/s10571-020-00955-7 PMC11441193 32876899

[130] Viscomi C Zeviani M. MtDNA-maintenance defects: syndromes and genes. J Inherit Metab Dis (2017) 40:587–99. doi: 10.1007/s10545-017-0027-5 PMC5500664 28324239

[131] Luciani A Denley MCS Govers LP Sorrentino V Froese DS. Mitochondrial disease, mitophagy, and cellular distress in methylmalonic acidemia. Cell Mol Life Sci (2021) 78:6851–67. doi: 10.1007/s00018-021-03934-3 PMC8558192 34524466

[132] Seranova E Connolly KJ Zatyka M Rosenstock TR Barrett T Tuxworth RI. Dysregulation of autophagy as a common mechanism in lysosomal storage diseases. Essays Biochem (2017) 61:733–49. doi: 10.1042/EBC20170055 PMC5869865 29233882

[133] Rajas F Labrune P Mithieux G. Glycogen storage disease type 1 and diabetes: learning by comparing and contrasting the two disorders. Diabetes Metab (2013) 39:377–87. doi: 10.1016/j.diabet.2013.03.002 23643353

[134] Melis D Rossi A Pivonello R Salerno M Balivo F Spadarella S. Glycogen storage disease type ia (GSDIa) but not glycogen storage disease type ib (GSDIb) is associated to an increased risk of metabolic syndrome: possible role of microsomal glucose 6-phosphate accumulation. Orphanet J Rare Dis (2015) 10:91. doi: 10.1186/s13023-015-0301-2 26219379 PMC4518509

[135] Rossi A Simeoli C Salerno M Ferrigno R Della Casa R Colao A. Imbalanced cortisol concentrations in glycogen storage disease type I: evidence for a possible link between endocrine regulation and metabolic derangement. Orphanet J Rare Dis (2020) 15:99. doi: 10.1186/s13023-020-01377-w 32306986 PMC7169016

[136] Rossi A Ruoppolo M Formisano P Villani G Albano L Gallo G. Insulin-resistance in glycogen storage disease type ia: linking carbohydrates and mitochondria? J Inherit Metab Dis (2018) 41:985–95. doi: 10.1007/s10545-018-0149-4 29435782

[137] Bhattacharya K. Dietary dilemmas in the management of glycogen storage disease type I. J Inherit Metab Dis (2011) 34:621–9. doi: 10.1007/s10545-011-9322-8 21491105

[138] Dahlberg KR Ferrecchia IA Dambska-Williams M Resler TE Ross KM Butler GL. Cornstarch requirements of the adult glycogen storage disease ia population: a retrospective review. J Inherit Metab Dis (2020) 43:269–78. doi: 10.1002/jimd.12160 31415093

[139] Qian H Chao X Williams J Fulte S Li T Yang L. Autophagy in liver diseases: a review. Mol Aspects Med (2021) 82:100973. doi: 10.1016/j.mam.2021.100973 34120768 PMC9585624

[140] Halaby CA Young SP Austin S Stefanescu E Bali D Clinton LK. Liver fibrosis during clinical ascertainment of glycogen storage disease type III: a need for improved and systematic monitoring. Genet Med (2019) 21:2686–94. doi: 10.1038/s41436-019-0561-7 31263214

[141] Gautam S Zhang L Lee C Arnaoutova I Chen HD Resaz R. Molecular mechanism underlying impaired hepatic autophagy in glycogen storage disease type ib. Hum Mol Genet (2023) 32:262–75. doi: 10.1093/hmg/ddac197 PMC10148728 35961004

[142] Busch V Gempel K Hack A Müller K Vorgerd M Lochmüller H. Treatment of glycogenosis type V with ketogenic diet. Ann Neurol (2005) 58:341. doi: 10.1002/ana.20565 16049943

[143] Vorgerd M Zange J. Treatment of glycogenosys type V (McArdle disease) with creatine and ketogenic diet with clinical scores and with 31P-MRS on working leg muscle. Acta Myol myopathies cardiomyopathies Off J Mediterr Soc Myol (2007) 26:61–3. PMC2949316 17915573

[144] Løkken N Hansen KK Storgaard JH Ørngreen MC Quinlivan R Vissing J. Titrating a modified ketogenic diet for patients with McArdle disease: a pilot study. J Inherit Metab Dis (2020) 43:778–86. doi: 10.1002/jimd.12223 32060930

[145] Similä ME Auranen M Piirilä PL. Beneficial effects of ketogenic diet on phosphofructokinase deficiency (Glycogen storage disease type VII). Front Neurol (2020) 11:57. doi: 10.3389/fneur.2020.00057 32117019 PMC7010930

